# Core dimensions of human material perception

**DOI:** 10.1073/pnas.2417202122

**Published:** 2025-03-05

**Authors:** Filipp Schmidt, Martin N. Hebart, Alexandra C. Schmid, Roland W. Fleming

**Affiliations:** ^a^Experimental Psychology, Justus Liebig University, Giessen 35394, Germany; ^b^Center for Mind, Brain and Behavior, Universities of Marburg, Giessen, and Darmstadt, Marburg 35032, Germany; ^c^Department of Medicine, Justus Liebig University, Giessen 35390, Germany; ^d^Vision and Computational Cognition Group, Max Planck Institute for Human Cognitive and Brain Sciences, Leipzig 04103, Germany; ^e^Laboratory of Brain and Cognition, National Institute of Mental Health, Bethesda, MD 20814

**Keywords:** vision, material perception, computational model, categorization, feature space

## Abstract

Visually categorizing and comparing materials is crucial for everyday behavior, but what organizational principles underlie our mental representation of materials? Here, we used a large-scale data-driven approach to uncover core latent dimensions of material representations from behavior. First, we created an image dataset of 200 systematically sampled materials and 600 photographs (STUFF dataset, https://osf.io/myutc/). Using these images, we next collected 1.87 million triplet similarity judgments and used a computational model to derive a set of sparse, positive dimensions underlying these judgments. The resulting multidimensional embedding space predicted independent material similarity judgments and the similarity matrix of all images close to the human intersubject consistency. We found that representations of individual images were captured by a combination of 36 material dimensions that were highly reproducible and interpretable, comprising perceptual (e.g., grainy, blue) as well as conceptual (e.g., mineral, viscous) dimensions. These results provide the foundation for a comprehensive understanding of how humans make sense of materials.

## Significance

Accurate perception of material properties is crucial for successful interactions with objects and substances in our environment. Materials such as wool, soil, skin, granite, and jelly have complex and highly varied appearances. Our ability to categorize and recognize them presumably relies on a set of properties, or “dimensions” that describe their characteristics, yet it has remained unclear which dimensions govern our mental representations of materials. Our study provides a systematic and highly comprehensive mapping of core dimensions underlying similarity judgments of materials. The results have broad implications for our understanding of material perception and open the door to a wealth of studies on how behavioral representations emerge and are reflected in the human brain.

A large part of the human cortex is dedicated to the processing of visual signals. In recent years, there has been tremendous progress in identifying the cognitive and neural mechanisms that determine how we see. While much of this research has studied the perception of objects—the things in the world around us (1–4)—recent work has shown that the human visual system is also highly tuned to processing materials (e.g., 5–7)—the *stuff* that things and the world are made of (8–10). Being able to accurately perceive material properties is crucial for interacting with our surroundings, whether we are judging if a lake is frozen, an apple is rotten, or a tool is wet and slippery.

**Fig. 1.**
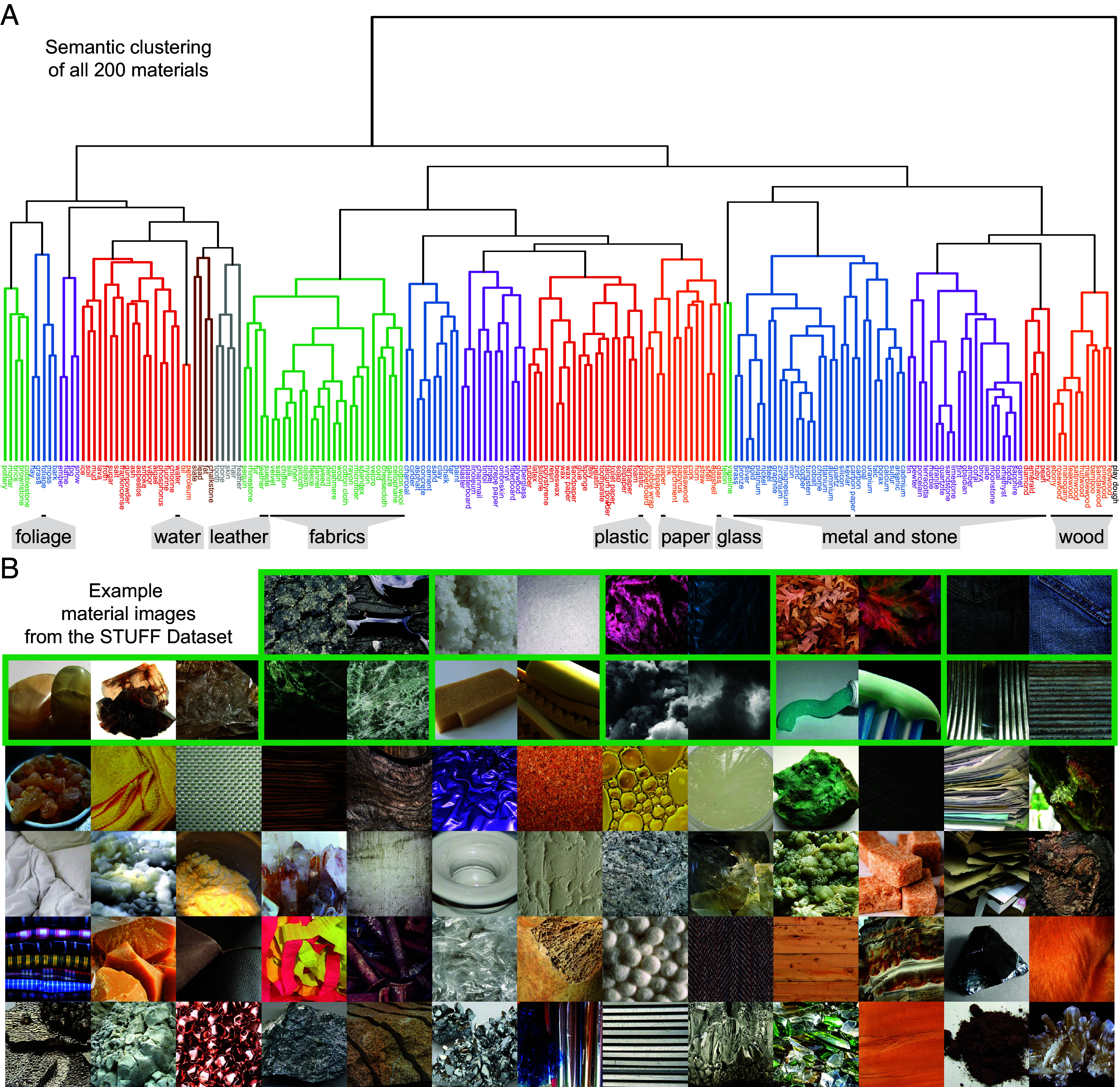
STUFF dataset. (*A*) Hierarchical clustering of all 200 material concepts based on an off-the-shelf semantic embedding for material nouns (11), illustrating the scope of our dataset. An approximate assignment of materials to the 10 superclasses of the Flickr Material Database (6) is shown below the dendrogram. (*B*) Examples from the 600 images of our STUFF dataset (https://osf.io/myutc/) (12), which contains 3 images per each of the 200 material classes; images from the same class are grouped by green frames. Copyright information for all images is provided in *SI Appendix*, Table S2.

Given the complex physical characteristics of natural materials and their enormous variability in visual appearance and functional significance (Fig. 1), how do we identify, compare, and categorize materials and, more generally, make sense of them so that we can interact with them in a meaningful manner? What underlying representations organize the wide range of materials and their properties to support everyday judgments and tasks?

A powerful way to characterize the mental representations of materials is in terms of material *properties*, or dimensions, paralleling previous work using objects (13). For example, it has been shown that different classes like water and wood can be described by a particular combination of material properties (14, 15): Water is high in perceived glossiness and transparency but low in roughness and hardness, while the opposite is true for wood. These studies hint at a mapping between dimensions of material perception and our ability to identify, categorize, and understand them, thus informing our everyday behavior in relation to materials. These material dimensions could encompass information about specific visual characteristics (e.g., surface color or texture properties), or information about conceptual characteristics (e.g., heavy or fragile).

However, while previous studies have revealed many important details of how properties and categories are inferred (15–17), they typically focused on small numbers of manually selected properties and restricted sets of materials, which may not generalize well to the wealth of material properties in our world. Other work successfully trained supervised deep neural networks to recognize materials or material properties from images or videos, or analyzed larger datasets of computer-generated images, or even depictions of materials in paintings (18–23). This, however, did not allow relating patterns of network activations to real-world perceptual features. And while it is possible to come up with a practically infinite number of candidate material dimensions, our ability to identify representational dimensions that underlie our mental representations of materials and are relevant for behavioral judgments is still surprisingly limited.

Here, we sought to gain a comprehensive and principled understanding of the properties that describe the complexity of material representations by identifying core dimensions that determine similarity judgments between materials. Similarity judgments offer an established approach to characterizing the multidimensional space underlying mental representations (24–26) and have been central to gaining access to the mental representations of objects (3, 4). It is thus plausible that similar approaches could benefit our understanding of material perception. To provide a comprehensive characterization of perceived material similarity, we first compiled a broad dataset of 600 natural material images, derived from 200 picturable material concepts in the English language. By systematically distilling those concepts based on predefined criteria from a comprehensive list of concrete English nouns, we made sure to cover very diverse and distinct materials. Next, we assessed the perceived similarity of these images in a large-scale crowdsourcing experiment comprising 1.87 million trials, in which we asked participants which of two material images was more similar to a third reference material image. In this paradigm, the nonselected material image acts as a context, which effectively highlights the relevant dimensions shared by the reference image and the selected one (27, 28). Based on a computational model of this task, we then identified a set of representational dimensions that characterizes material similarity judgments.

The dimensions of our model captured ~90% of variance in perceived similarity with only 36 dimensions. These dimensions were generally interpretable and encompassed aspects of the materials’ appearance (e.g., texture, shape, and color), mechanical properties (e.g., viscous) as well as membership of certain material classes (e.g., metal, wood). Some of the discovered dimensions matched those that previous experimenters have investigated, providing a data-driven, empirical basis for their role in the representation of materials [texture and color (14, 15, 17)]. Other dimensions did not feature in earlier research, for example, “crystalline,” “small,” “spongy,” “thin,” or “hot”. Our findings and material embedding have broad application for studying material perception, its natural dimensions, and the representation of material features and categories in the human brain.

## Results

We aimed to uncover representational dimensions underlying similarity judgments of materials by taking a data-driven approach, where we 1) created a broad, systematically sampled dataset of material images, 2) collected a large-scale crowdsourced behavioral similarity dataset for these images, and 3) carried out computational modeling to identify core dimensions underlying these similarity judgments.

### STUFF: A Dataset of 200 Material Classes in 600 Images.

First, we sought a systematic method for identifying material categories to mirror the full richness and complexity of material appearances in the real world. To this end, we identified an extensive list of material words, as word usage captures many of the behaviorally relevant distinctions between different material classes (29, 30). Specifically, we started with 8,671 concrete nouns in the American English language, which were distilled into a comprehensive set of picturable material concepts according to a predefined set of criteria (for a detailed description of the selection criteria see *Materials and Methods* and *SI Appendix*, *Supplementary* *Materials and Methods*). This procedure yielded 200 distinct concepts spanning materials as diverse as algae, brass, ebony, fleece, oil, rubber, and zinc (see *SI Appendix*, Table S1 for a complete list). The resulting list of 200 materials is much shorter than comparable lists of objects (29, 30), yet far longer than the number of material classes considered by previous studies in material perception (14, 21, 31). This indicates that the diversity of material names may be lower than that for objects, while highlighting that broad, systematic sampling can lead to a much wider range of material classes than previously studied.

The scope of the dataset can be visualized by clustering all materials based on their semantic similarity, using correlations between 300-dimensional sense embedding vectors for each material noun (11). This embedding is an extension of Word2Vec, (32), which is a word embedding, to WordNet synsets (cf., *SI Appendix*, Table S1), which is a sense embedding and is thus expected to be more precise. The resulting semantic clustering illustrates the broad semantic diversity of the materials (Fig. 1*A*). This is also supported by the fact that while our materials include WordNet hyponyms of all 10 superclasses of the influential Flickr Material Database (6) (e.g., stone, glass, water), about half of our materials (106 out of 200) extended beyond all of these superclasses.

Having verified the semantic breadth of the 200 materials, we went on to collect three high-quality, close-up, naturalistic photographs of each material concept. Specifically, we performed an extensive web search for materials depicted in their typical aggregate state and form (e.g., liquid oil or grains of salt), including close-ups of object surfaces, leading to a focus on the material rather than the object. The resulting 600 images of materials were cropped to square aspect ratio (Fig. 1*B*). The complete STUFF dataset, and an extended version with 15 images per concept, is available for download from https://osf.io/myutc/ (12).

### A 36-Dimensional Representational Embedding Captures Single-Trial Material Similarity Judgments.

**Fig. 2.**
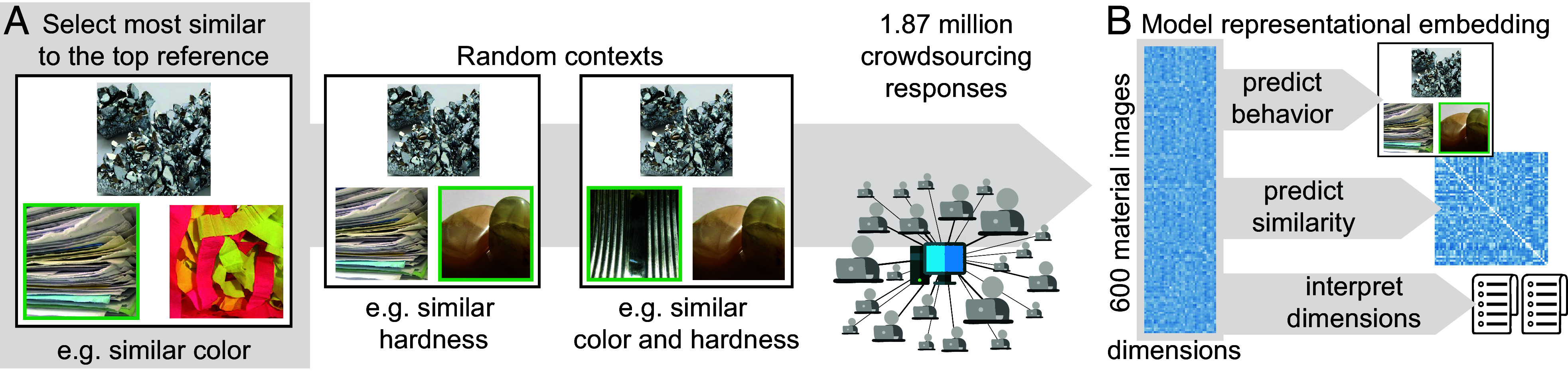
Experimental paradigm and modeling. (*A*) Illustration of the triplet 2-AFC task. Material images were presented to participants in different contexts imposed by the third material in a triplet. We used online crowdsourcing to sample across a wide range of these random contexts. (*B*) The goal of the modeling procedure was to learn a representational embedding (28) that i) captures choice behavior in the triplet 2-AFC task, ii) predicts similarity across all pairs of materials, and iii) provides interpretable material dimensions. Since only a subset of all possible triplets had been sampled, the model also served to estimate the complete similarity matrix. Copyright information for all images is provided in *SI Appendix*, Table S2.

Having assembled a broad dataset of material classes and images, we next sought to measure the perceived similarity between the 600 images in the STUFF dataset, in a triplet two-alternative forced choice (2-AFC) task. In a given trial, we presented a reference material image in a triangular arrangement with two other test materials and asked participants to identify which of the bottom two images was more similar to the top reference image (19). In this task, we defined similarity as the probability of choosing two material images together, marginalized across all contexts imposed by the third image. For example, participants judged chrome as similar to moonstone when compared to paper, but as dissimilar to moonstone when compared to tin (Fig. 2*A*). Depending on the context, different properties—such as color, hardness, or gloss—could dominate the decision. As a consequence, the triplet task highlights the relevant dimensions that form the basis of similarity judgments. By sampling very broadly across many such contexts, we can thus determine the similarity of two images according to a broad range of possible material dimensions.

For the triplet 2-AFC task, a full sample of the 600 × 600 similarity matrix would require a total of ~107.46 million trials. Given the excessive cost associated with acquiring a full similarity matrix, and since we anticipated a similarity matrix with a much lower rank than the number of images, we instead collected a smaller subset of 1.87 million responses (1.74% of all possible unique triplets) from a sample of 5,038 workers on the online platform Amazon Mechanical Turk. We then used a computational procedure modeling the underlying decision process (see below), which allowed us to fill the gaps in the similarity matrix. To estimate the intersubject rating consistency and thus the best possible performance any pooled-participant model could achieve given the data, part of this dataset was a separate, randomly chosen set of 1,200 triplets which we sampled 60 times each. To test how well a model based on our sample can reproduce the full similarity matrix, we also collected a separate sample of all triplet similarity relations for a subset of 60 images, with each possible triplet repeated twice (205,320 responses). All relevant code and deidentified data are available for download from https://osf.io/5gr73/ (33).

To model the decision process underlying the similarity judgments, we sought a computational modeling approach that could: 1) predict behavioral responses from the triplet 2-AFC task, 2) generalize to all pairs of material images in the dataset, and 3) provide interpretable material dimensions. To achieve these objectives, we used the sparse positive similarity embedding technique described previously (27, 28) (code available at https://github.com/ViCCo-Group/SpoSE) adapted to a 2-AFC task and quantified material images as vectors in a multidimensional representational space (Fig. 2*B*).

Specifically, the full representational embedding constitutes a matrix in which columns correspond to material dimensions and rows to material images. Each row thus describes a material image as a vector in a multidimensional feature space. The representational embedding is built on three key assumptions about the material dimensions: sparsity, continuity, and positivity. The sparsity assumption reflects the fact that not all features are expressed in all materials (e.g., marble would score zero on a putative dimension of viscosity). Continuity and positivity assumptions allow us to interpret the nonnegative numerical value for a given dimension as the degree to which that feature is expressed in a material (e.g., toothpaste is higher in viscosity than water) (34). These constraints thus yield dimensions that represent behaviorally relevant parts of images and have been shown to be meaningful to other human observers (28, 35). The model embedding was initialized with 90 dimensions composed of random numbers and was trained on 90% of available trials, with the remaining 10% serving as an independent test set. To induce sparsity, the model was regularized with an L1 norm, and the regularization parameter λ—which controls the trade-off between sparsity and out-of-sample model performance—was determined using cross-validation on the training set (λ = 0.0038).

**Fig. 3.**
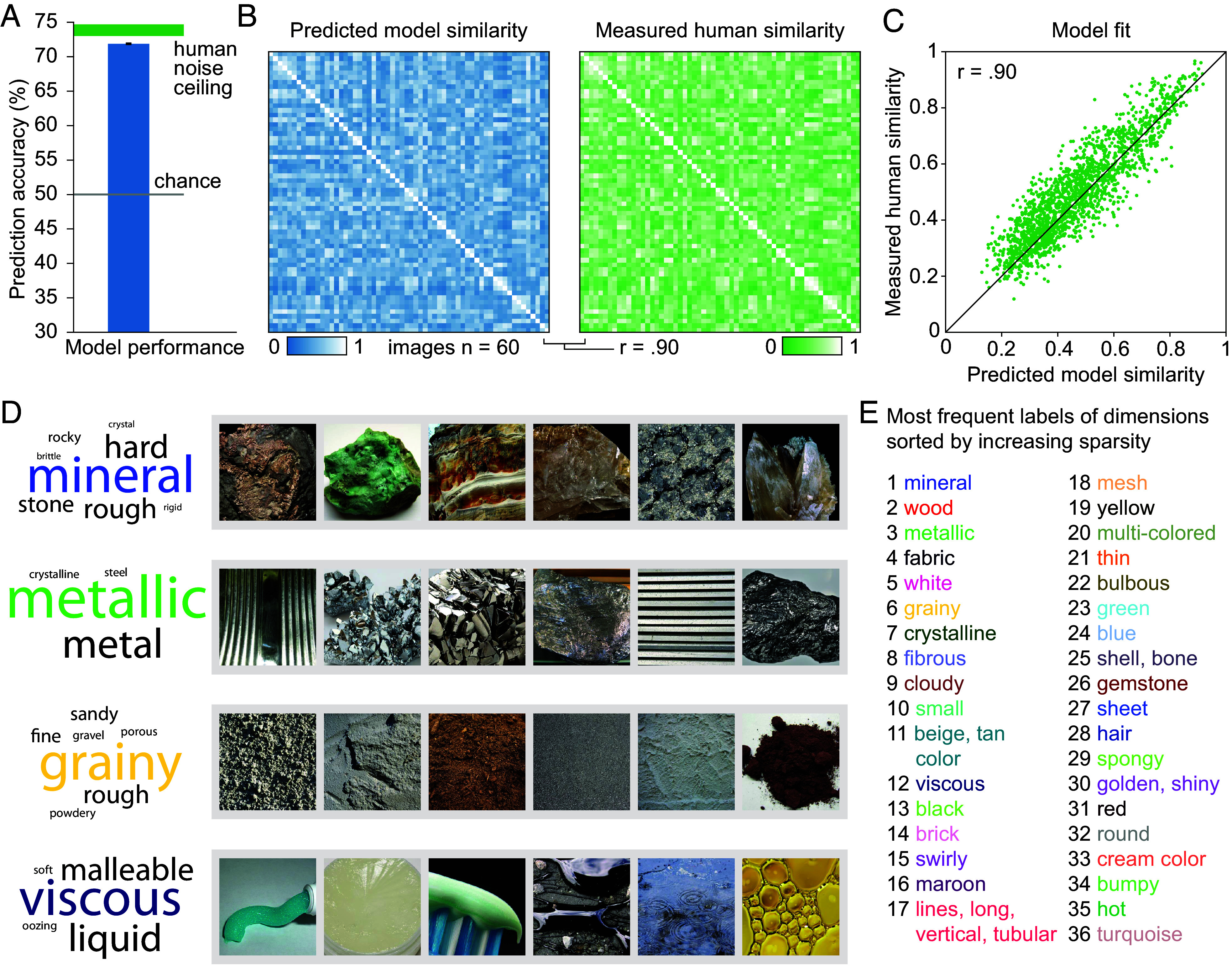
Modeling results and interpretability of model dimensions. (*A*) Model prediction performance for individual trials in independent test data, relative to chance (gray) and the human intersubject consistency (green). The intersubject consistency denotes maximal performance given the between-participant variation which is due to noise or true differences between participants and is obtained by calculating the consistency in participants’ responses to the same triplet. The model reached 91.7% of the intersubject consistency. Error bar for prediction and width of intersubject consistency bar denote 95% CI. (*B*) To estimate how well the model predicted behavioral similarity, we compared a fully sampled behavioral similarity matrix for a subset of 60 images (blue) to the model-generated similarity matrix for these images (green). (*C*) The close fit between both shows that most explainable variance was captured by the model embedding (Pearson’s r = 0.90; *P* < 0.001; randomization test; 95% CI, 0.88 to 0.91). (*D*) Visualization of four example dimensions and associated results of the dimension-labeling experiment. The example dimensions are visualized showing six images with large embedding weights in these dimensions. The word clouds reflect a summary of the semantic labels provided by participants for these dimensions (here only showing labels mentioned more than once; for the complete word clouds for all dimensions see *SI Appendix*, Fig. S4). Copyright information for all images is provided in *SI Appendix*, Table S2. (*E*) The most frequent labels provided for each dimension, with dimensions 1 to 36 ordered according to their sparsity (i.e., mineral showed the lowest sparsity with almost all of our 600 images having nonzero values; turquoise color showed the highest sparsity with only a few images having nonzero values).

We iteratively adapted the weights of the 90 dimensions based on the difference between the model’s predicted choice probability and the empirically measured choice. At the end of training, as a result of the sparsity constraint, 54 dimensions yielded values consistently close to 0 and were eliminated (*Materials and Methods*). The resulting embedding thus contained 36 dimensions, which we sorted on the basis of the sum of all dimension values averaged across all materials, in descending order (Fig. 3).

Due to the stochastic nature of the modeling procedure, fitting the model repeatedly may lead to a different embedding and a slightly different number of dimensions. Thus, to estimate the stability of the embedding, we ran it 50 times with different random initializations (*Materials and Methods*). Across those embeddings, most dimensions were highly reproducible (Pearson’s r > 0.90 in 28/36 dimensions and r > 0.8 in 34/36 dimensions), indicating the stability of our identified embedding (see *SI Appendix*, Fig. S1 *A* and *B* for the reproducibility of all dimensions). To test the stability across image subsets, we randomly removed 18% of the categories and ran 50 random initializations with these subsets. Although this greatly reduced the training set by removing 44.90-44.97% of triplets, the overall reproducibility of dimensions only decreased slightly by r = 0.07 on average (*SI Appendix*, Fig. S2*A*). Dimensions that were less reproducible across random initializations were also less stable across image subsets (r = 0.90, *P* < 0.001). Overall, this demonstrates that most dimensions were highly stable across initializations or image subsets, highlighting that the embedding is not dependent on idiosyncrasies of individual images in the dataset.

To evaluate the embedding’s predictive performance for triplet 2-AFC judgments, we computed the human intersubject choice consistency from the additional repeated sample of 1,200 randomly chosen triplets (see above). Averaged across all these triplets, given the variance in responses, the theoretical upper limit for fitting individual trials from the data was 73.84% (chance = 50%), with the model predicting 71.86% of individual trials in the independent test data. Thus, with respect to the best possible prediction any between-participant model could achieve (human intersubject consistency), the model reached a chance-corrected performance of 91.70% at the individual-trial level (Fig. 3*A*). To underscore the sensitivity of this approach to subtle changes in choice probability, we confirmed this prediction by fitting the predicted choice probability for the 1,200 intersubject consistency triplets to the actual choice probabilities, yielding a predictive accuracy of 90.25% and a correlation coefficient of r = 0.81 (*SI Appendix*, Fig. S3).

### Accurate Reconstruction of Perceived Material Similarity Judgments.

Having confirmed that the embedding could accurately predict individual trial behavior, next we evaluated how well it could predict behaviorally measured similarity. To this end, we compared the fully sampled similarity matrix derived from the random sample of 60 images with the predicted similarity using the 36-dimensional representational embedding (Fig. 3 *B* and *C*). The matrices were highly correlated (Pearson’s r = 0.90; *P* < 0.001; randomization test; 95% CI, 0.89 to 0.91). To reveal how well this prediction worked as a function of the noise in the data, we measured the split-half reliability of the fully sampled similarity data of the 60 images. The split-half reliability of the similarity matrix was r = 0.97 (Spearman–Brown corrected), showing that we were able to predict 83.31% of the explainable variance in similarity. These results demonstrate that despite a large variety of visual appearances in the dataset and many possible features that can contribute to material judgments, a low-dimensional representational embedding was able to accurately reproduce behaviorally measured similarity for material images.

To better understand the structure of the similarity matrix, we further examined the structure of the predicted 600 × 600 similarity matrix. As expected by the 2-AFC task, we observed a mean similarity of 0.50, with a wide spread of similarities (SD = 0.18, range = 0.11 to 0.98). Similarities within each of the 200 material classes (M = 0.84) were higher than similarities between different material classes (M = 0.50, t(199) = 45.28, *P* < 0.001). Specifically, similarity was highest between images within material classes of brick, straw, and lava (all 0.97) and lowest within classes of fleece, feather, and onyx (0.63, 0.61, and 0.60). Thus, even though our dataset contained multiple examples per material class, participants’ responses to images within each class still exhibited a high degree of variability, likely due to differences in material appearance.

Given the semantic structure present in the STUFF dataset as revealed by the validation analysis (Fig. 1*A*), it may be expected that semantics would dominate the behavioral judgments (36). Indeed, distributional word vector models in the past have been shown to correlate well with human word similarity judgments, explaining up to 50% of their variance (e.g., 37). Therefore, we obtained the similarity matrix for material nouns from correlations between their individual 300-dimensional semantic embedding vectors (11, 32) and correlated the resulting semantic similarity matrix to our behavioral similarity matrix. We found a small effect size (R2 = 0.11), suggesting that a substantial amount of variance in behavioral similarity is explained by factors other than conceptual knowledge captured in semantic embeddings (e.g., visual appearance).

To test the degree to which the representational embedding could predict each of the 200 material classes, we iteratively trained a linear support vector machine on just 2 examples on the 36-dimensional embedding and evaluated it on the left-out third example. This yielded a pairwise accuracy of 97.21% (chance = 50%), and top-1 and top-5 accuracies of 47.00% and 74.33%, respectively (chance levels: 0.5 and 2.5%, respectively; median rank of correct answer: 2). Thus, the representational embedding is highly informative about material classes, despite being based on only 36 dimensions and using only two training examples, reinforcing the notion that representations of materials derived from their visual percept are substantially richer than purely semantic representations derived from words.

### Interpretable Dimensions of Material Representation.

To determine the nature of the representational dimensions underlying material similarity judgments, we next sought to test whether the 36 dimensions in the embedding were interpretable. To this end, we first asked a separate group of observers (n = 20) to provide verbal labels for each dimension. On each trial, we visualized a given dimension by showing material images that spanned a broad range of feature values, ranging from images with high weights on these dimensions to images with low weights. Observers then entered verbal labels to describe the depicted characteristic (*SI Appendix*, *Supplementary Materials and Methods*). This provided us with semantic labels for each of the 36 dimensions (Fig. 3*D*).

For all participants combined, we obtained an average of 40.40 labels for each dimension (mean = 2.02 labels per participant; range = 1 to 10 labels), with good agreement considering the underconstrained task: In each category, the three most frequent labels together accounted for an average of 81% (range: 15 to 85%) of observers’ responses. Labels were conceptual (e.g., “mineral”), or referred to optical (e.g., “metallic”), texture (e.g., “grainy”), shape (e.g., “round”), or physical properties (e.g., “viscous”) (Fig. 3*D*). Material color was shown to be related to a sizeable fraction of dimensions, highlighting the importance of color for discriminating among materials (5, 38–41). All labels with >20% agreement in the naming task are provided in *SI Appendix*, Table S3. By identifying dimensions by their most frequent label and sorting them according to their sparsity, we can show to what extent different features explained variability in the behavioral data (Fig. 3*E*). In addition to this labeling approach, to determine the interpretability without potential rating bias, we pursued a separate, data-driven approach based on identifying semantic features that are shared between materials loading strongly on individual dimensions (*SI Appendix*, *Supplementary Materials and Methods* and Table S4), which for dimensions not purely defined on image characteristics (e.g., mineral) closely mirrored the results found in participants.

**Fig. 4.**
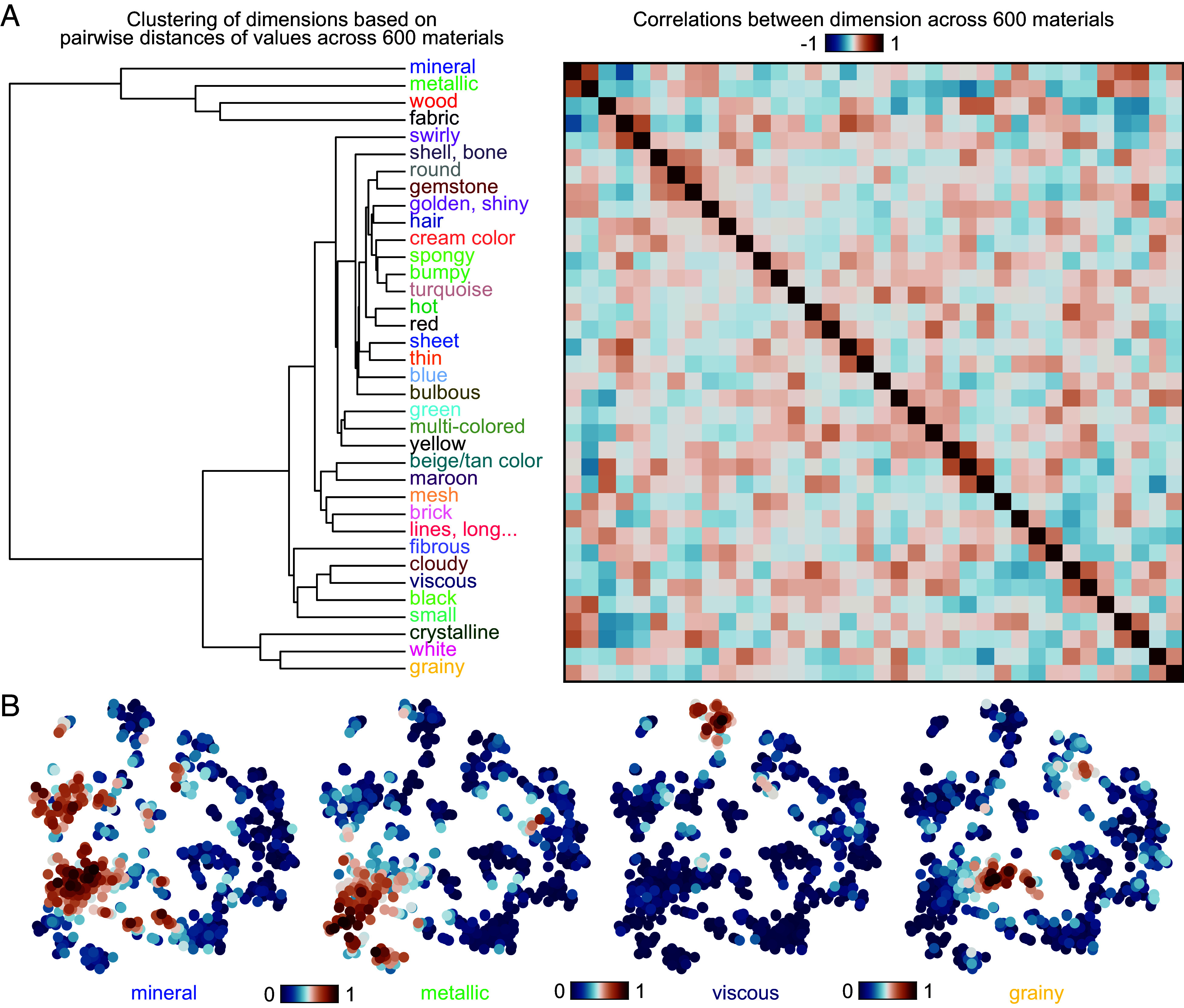
Similarity between model dimensions and expansion in material similarity space. (*A*) Clustering of 36 model dimensions based on the pairwise distances between their values across all 600 materials, together with the correlation matrix, showing mostly low to moderate correlations between dimensions (mean correlation r = −0.01, SD = 0.12, range = −0.51 to 0.37). (*B*) The distribution of weights for four example dimensions across all 600 images, visualized by plotting images as points in a two-dimensional t-SNE visualization of the similarity embedding (initialized with multidimensional scaling; dual perplexity, 5 and 30; 1,000 iterations). Color represents how strongly each image expressed the particular dimension (normalized to range 0 to 1: blue–red).

To characterize the relationship between the 36 model dimensions, we conducted hierarchical clustering of dimensions across all material images (Fig. 4*a*). This analysis highlights what dimensions are coexpressed in material images. For example, the dimension “gemstone” formed a cluster with the dimension “round,” indicating that gemstone materials tend to be round. Likewise, the dimension “hot” formed a cluster with the dimension “red,” and the dimension “brick” with the dimension “lines, long, vertical, tubular,” highlighting other important commonalities between material classes. Dimensions that covaried also extended across corresponding areas of the material similarity space that we visualized by projecting to two dimensions using t-SNE, with some dimensions overlapping in materials (mineral, metallic) and others hardly ever (mineral, viscous; Fig. 4*B*). However, correlations between the 36 dimensions were generally low (maximum Pearson’s r = 0.37), with only two dimension pairs showing a moderate negative correlation (mineral vs. fabric, r = −0.51; metallic vs. beige, r = −0.41). The overall distinctiveness of dimensions is also apparent in the sparsely localized maps of high feature values in Fig. 4*B*. Together, these results reveal which dimensions materials tend to have in common while highlighting that dimensions reflect genuinely distinct material attributes.

The distribution of material classes used for training the similarity embedding presumably affected the dimensions we discovered. For example, in the STUFF dataset, of the 10 superclasses in the Flickr Material Database (6), a semantic cluster of 56 metal and stone materials was represented more frequently than other superclasses (Fig. 1*A*). Yet we found that the impact of this was surprisingly small. We repeated the model fitting procedure 50 times while removing 36 metal or stone materials (18% of all material categories) and estimated the dimension stability in comparison to the removal of random materials described above (*SI Appendix*, Fig. S2*B*). The results revealed a highly similar pattern to random removal (see above). In addition, as expected, dimensions specifically related to metal or stone materials were slightly more affected (e.g., dimension “metallic” or “golden, shiny”), indicating only small effects specific to the frequency of material categories. Together, this demonstrates that the distribution of material classes in STUFF likely only slightly affected the dimensions discovered with our data-driven embedding approach.

### Visualizing and Interpreting Dimension Profiles for Individual Images and Global Similarity.

**Fig. 5.**
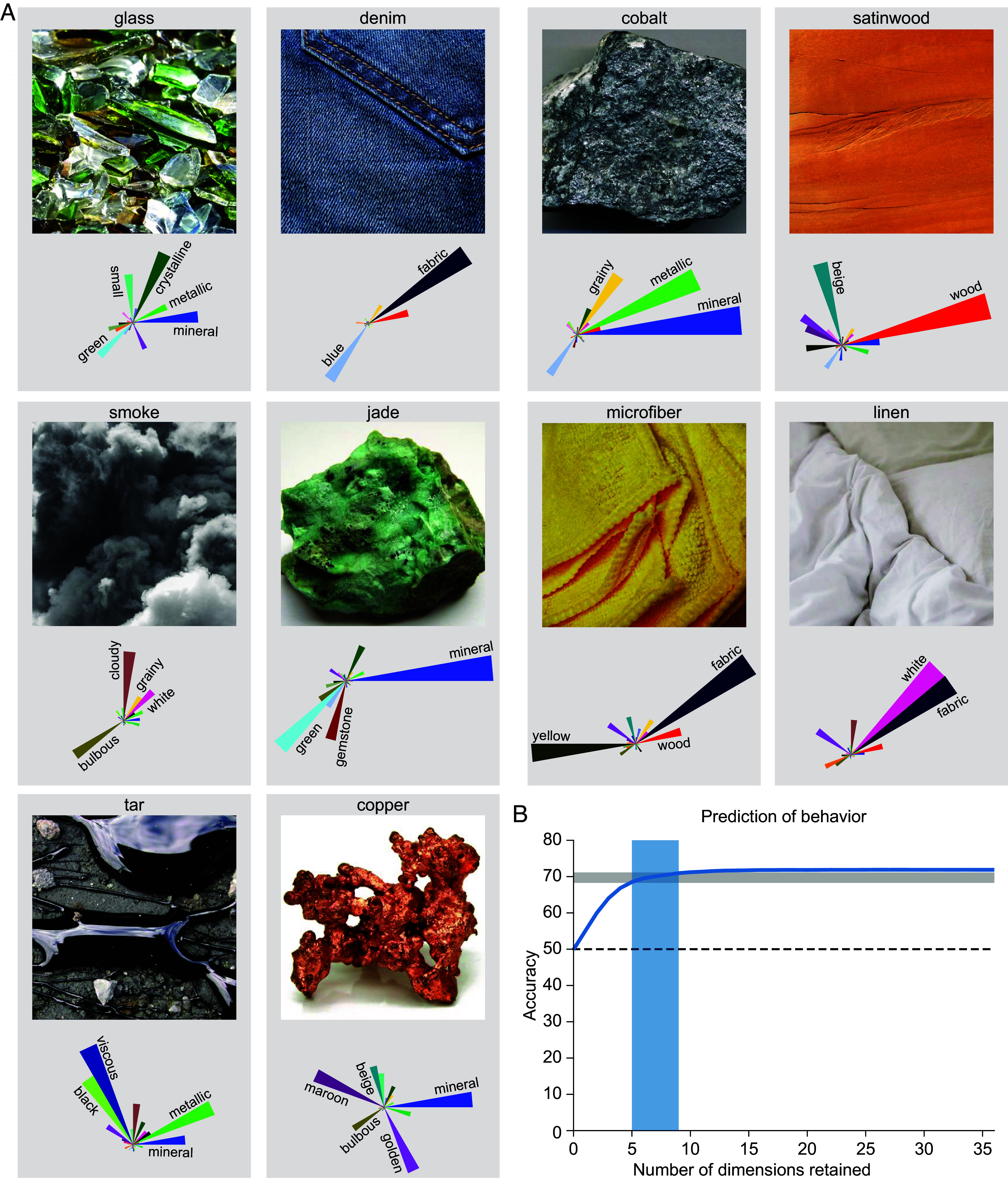
Behavioral judgments and similarity for individual images are well explained by 5 to 9 dimensions. (*A*) Example material images and corresponding distributions across dimensions, using rose plots with each petal reflecting the degree a material dimension is expressed for that image. Petal orientation and colors indicate individual dimensions and length indicates the value in that dimension. Dimension labels are only provided for weights > 0.53. Copyright information for all images is provided in *SI Appendix*, Table S2. (*B*) For explaining 95 to 99% of the predictive performance in behavior, only 5 to 9 dimensions per image are required; however, these dimensions varied between images (see main text for details).

Having characterized the interpretability of the 36 dimensions across all materials, we further explored the dimensions’ interpretability by visualizing dimensions for individual images (Fig. 5*A*). For instance, the example image of smoke is characterized primarily by the dimensions of “cloudy,” “bulbous,” “white,” and “grainy,” while the image of jade is characterized primarily by the dimensions of “mineral,” “green,” and “gemstone”. The fact that each individual image can be described by a small number of dimensions suggests that not all 36 dimensions are required for all similarity judgments. To quantify this observation, we tested the predictive accuracy of the model by maintaining only the most prominent dimensions for each material. For this, we set the individual material’s dimension with the lowest weight to zero, predicted behavior, recomputed the similarity matrix to measure the effect on the model’s predictive performance, and repeated this process until only one dimension was left. This analysis revealed that in order to explain 95-99% of the variance in the raw triplet-task responses, only 5 to 9 dimensions were required in any given trial (Fig. 5*B*). Accordingly, human responses to individual material images indeed appear to be driven by the expression of a small number of decisive features, while at a global level, and across individuals, observers may integrate across a larger number of these dimensions, highlighting the importance of taking diverse features into account for judging the similarity of materials (28).

**Fig. 6.**
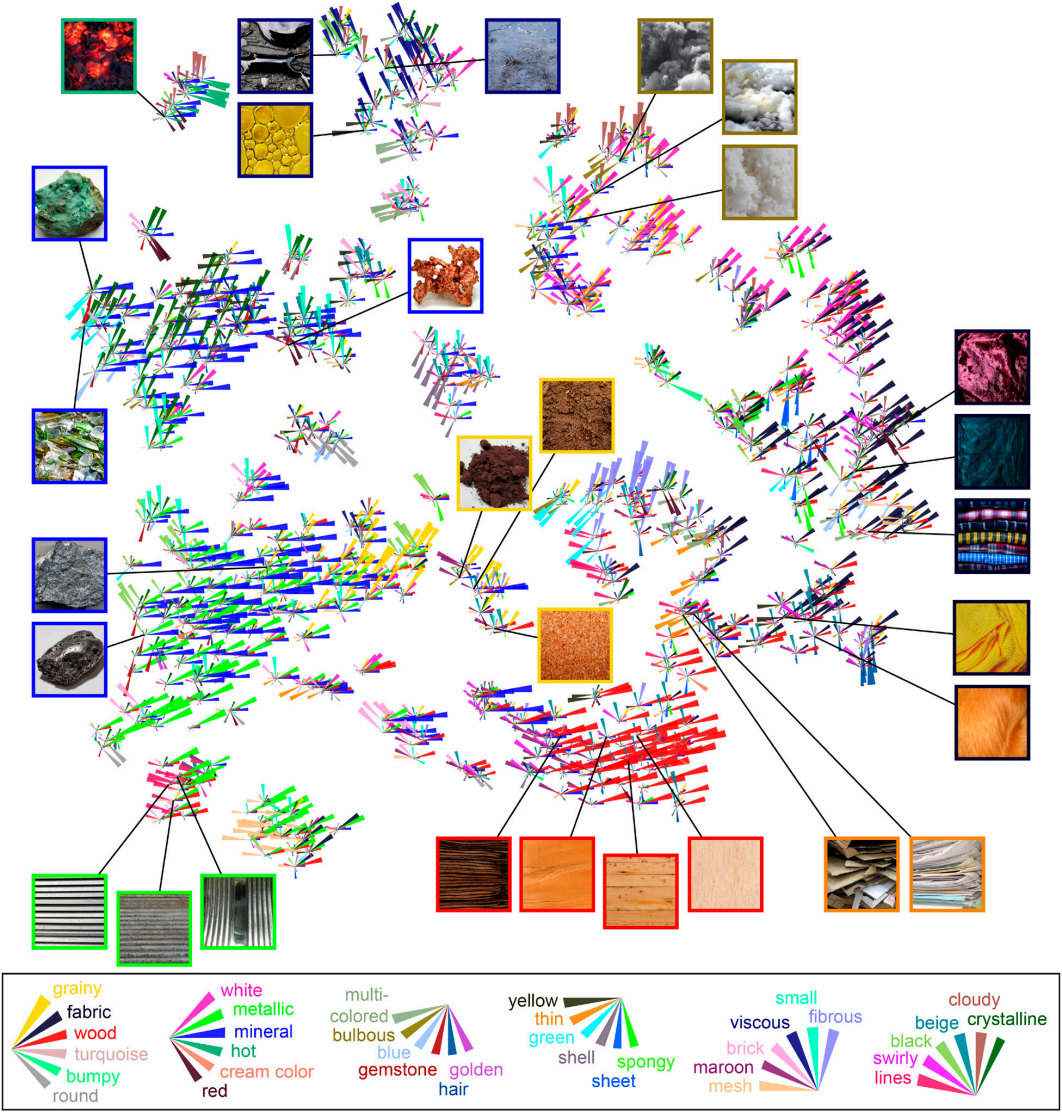
Two-dimensional visualization of the similarity embedding. The similarity embedding is visualized by combining rose plots for each material with t-SNE dimensionality reduction (initialized by multidimensional scaling; dual perplexity, 5 and 30; 1,000 iterations). Frames of example images are colored according to the dominant dimension, but note that multiple other dimensions also play a role for each stimulus. Copyright information for all images is provided in *SI Appendix*, Table S2.

Finally, to characterize the global similarity structure with respect to the 36 dimensions, we combined the visualization of dimension profiles for individual images with the t-SNE projection of the similarity embedding for all 600 material images (Fig. 6). The organization shows large clusters that reflect the expression of particular material properties (e.g., minerals, fabrics, woods, or viscous materials). However, the space is also highly organized within these larger clusters, with local arrangements of materials according to expressions of nondominant properties (e.g., different colors within minerals, fabrics, or viscous materials; *SI Appendix*, Fig. S5). At a global level, we note a gradient from images with bulbous and clumpy appearances (*Top Right*) to more linear parallel structures, like stripy wood grain or paper sheets (*Bottom*) via grainy and fibrous materials (*Middle*). Another notable gradient is the separation of hard materials (*Left* and *Bottom*) from softer materials (*Right*), with grainy and fibrous materials in between. Hot materials (*Top Left*) appear to be relative outliers in a tight cluster separated from the rest.

This visualization provides a more comprehensive and multifaceted view of the relationship between material properties and categories than previous studies. It is noteworthy that many of the material characteristics (e.g., grainy, fibrous, hot) are currently underrepresented in material perception research (36, 37), yet play an important role in the global organization of the perceptual representation of materials. Future research should investigate the visual cues underlying such characteristics. To further inform future investigations, we also analyze in more detail the relationship between our findings and object similarity judgments (*SI Appendix*, *Supplementary Materials and Methods*).

### An Image-Computable Model of Material Similarity Judgments.

Despite the overall predictive power of our approach and the stability of the learned embedding, our approach is inherently limited to the set of images the model has been trained on. To test the degree to which we can use the embedding to obtain similarities even for novel images without sampling behavioral judgments, we used an image-computable computational approach to generate similarities (42). Specifically, we computed the activations of the visual projection layer of the multimodal deep neural network CLIP-ResNet-50 × 64 (OpenAI training). From this, we used ridge regression to predict the values of individual material images in each of the 36 dimensions, using a separate leave-one-material-out procedure for each dimension and nested cross-validation to determine the best regularization parameter. Using these dimensions derived from the embedding, we modeled synthetic similarity judgments by computing all possible predicted odd-one-out choices. The resulting global similarity matrix showed a strong correlation with the similarity matrix predicted from the similarity embedding based on behavioral similarity judgments (r = 0.86; *SI Appendix*, Fig. S6). To identify the degree to which this result would generalize to novel images and to an empirically sampled similarity matrix, we collected additional similarity judgments for a sample of 48 novel material images picked from the extended set of STUFF images and applied the same procedure to these images. We chose the best hyperparameter from the training set. The comparison with predicted and human similarities continued to yield a high correlation (r = 0.79). This demonstrates that it is possible to combine deep neural networks with our behavioral data to predict dimension values and similarities for novel material images.

## Discussion

Our world is made of stuff—and our visual system is highly attuned to processing and interpreting that stuff (e.g., 5, 6). Material appearances vary enormously, both between and within material classes, making classifying and comparing materials computationally challenging. Our mental representation of materials must be robust and efficient, yet also sufficiently multifaceted to support diverse behaviors (8–10). Here, we sought to characterize such representations by moving beyond small-scale experimental approaches that inevitably rely on limited stimulus sets (14, 20, 21, 31) and tasks (43, 44) restricted by the goals of the experimenter, which therefore may not capture important aspects of our perceptual experience. Instead, we used a large-scale, data-driven approach to identify core dimensions underlying similarity judgments of materials. We broadly and systematically sampled 200 material classes, assembled 600 photographs of these materials exhibiting highly diverse characteristics and appearances, collected over 1.8 million perceptual similarity judgments between triplets of these photographs, and applied computational modeling methods to derive intuitively interpretable dimensions from behavioral responses. This revealed a set of 36 dimensions along which participants appear to judge similarity of material images, capturing diverse characteristics related to surface reflectance and texture properties (e.g., “green,” “repetitive”), shape-related properties (e.g., “thin,” “round”), physical attributes (e.g., “malleable,” “brittle”), and category membership (e.g., “wood,” “paper”). These 36 dimensions can account for the majority of the explainable variance in the similarity judgments, and thus provide the most comprehensive and systematic mapping of the representation of materials in the human visual and cognitive system to date. In addition, these findings provide a foundation for future studies of material representations and studies that map out how material dimensions are encoded in the human brain.

### Characterizing Mental Representations using Material Similarity Judgments.

The STUFF dataset covers 200 materials in 600 images, and we also provide an extended set for future studies with at least 15 images for each material. Our findings provide a proof-of-principle demonstration that material classes—as defined by linguistic terms—are an effective way to define a stimulus set that captures information about behaviorally relevant properties. Of course, alternative decisions about exactly which terms and images to include would have some impact on the findings. For example, the low sparsity of the mineral dimension likely reflects the fact that the STUFF dataset contains a lot of minerals, since there are many distinct nameable minerals (e.g., granite, jade, amethyst). As expected, including fewer mineral samples does indeed reduce the prominence of the “mineral” dimension, however, without affecting its presence. Indeed, we also found that the dimensions were highly robust against omitting significant numbers of (mineral) material categories and images. Future studies could consider the frequency of encounters with different material classes and their behavioral relevance when defining the dataset. Yet, despite this, our analyses suggest that the dimensions we have identified are surprisingly reproducible and robust, extending to behaviors beyond similarity judgments, such as categorization.

One reason for the robustness of our findings is that the semantic sampling is not strongly reflected in the similarity judgments. Instead, a substantial amount of variance in the similarity data was explained by factors other than conceptual knowledge captured in semantic embeddings. This suggests a key role of appearance characteristics such as the color, texture, and shape of the materials in driving similarity judgments. Visually inspecting the ranking of stimuli along individual dimensions (e.g., grainy) reveals clearly appearance-based organization.

### Advantages of Our Approach for Future Studies.

The modeling approach used here has a number of benefits. Most notable is the ability of the 36-dimensional representational embedding to accurately capture single-trial material similarity judgments (Fig. 3). This low dimensionality drastically reduces the complexity of relating features to external behavior and allows comprehensive modeling of interpretable brain signals in response to material dimensions. Another advantage of taking a data-driven approach is the possibility of discovery unconstrained by the experimenter’s hypotheses about relevant dimensions. The modeling approach also allows a way to infer a multidimensional representation, while recognizing that not all dimensions are expressed in all materials. Finally, by definition, the dimensions comprise properties whose distinction is behaviorally relevant—at least in terms of determining the perceived similarity between items. As revealed by the clustering analysis, the dimensions capture distinct aspects of material appearance; the embedding of items in the multidimensional space reveals which dimensions materials tend to have in common while highlighting that dimensions reflect genuinely distinct material attributes. We provide an image-computable model of material similarity judgments that can be used to predict dimension values and similarities of novel images from deep neural network activations.

### Interpretability and Selection of Dimensions.

The dimensions that emerge from our analysis reflect a general-purpose representation that can be selectively sampled for different tasks. Importantly, despite the relatively unconstrained task, the dimensions that emerged were highly interpretable by other participants, who tended to provide consistent labels for the dimensions: The three most frequent labels together accounted for an average of 81% of observers’ responses.

It is interesting to note that to explain 95 to 99% of the variance in the raw triplet-task responses, only 5 to 9 dimensions were typically required in any given trial. Accordingly, human responses to individual material images indeed appear to be driven by the expression of a small number of decisive features, while at a global level, and across individuals, observers may integrate across a larger number of these dimensions. This raises an interesting question about how observers so rapidly and efficiently identify decisive features for a given image out of a large number of possible dimensions. This is an example of a more general challenge for cognitive systems of how to determine key features of a single stimulus, for example in one-shot learning (45–47). We have speculated previously that salient or atypical image features could provide good candidates (48). But it is also noteworthy that feature activations tend to be quite sparse, with most features yielding negligible response for most images. Thus, the “decisive features” for a given trial might simply be those with nonnegligible activations across two of the three images.

Another important aspect of the dimensions is their continuous nature, which represents the degree to which a given feature is expressed in a material (e.g., toothpaste has higher viscosity than water). As the dimensions included both appearance attributes (e.g., color and texture), and categories (e.g., metal, wood), this provides a unification of perceptual qualities and material classes (14) within a single framework. Rather than expressing category membership as a simple binary quantity, the dimensions provide continuous representations of properties that reflect the degree to which individual images exhibit particular material properties and categories. Moreover, our clustering analysis revealed that the representational embedding is highly informative about material classes, despite being based on only 36 dimensions and using only two training examples per category.

While most dimensions reflected material properties, some dimensions also describe properties that not only relate to materials but also to other stimuli. Of note, color featured particularly prominent within our dimensions. This makes sense as color is an integral aspect of material perception (5, 38–41) and can even dominate material categorization. For example, color is more intricately connected to the identity of materials, in contrast to most objects: We often classify liquids based on color alone (water vs. wine) (39), whereas perceived object identity relies much more on shape features and is more invariant across changes in color.

### Limitations and Future Directions.

One of the main limitations of our dataset and results is that we did not consider “food materials” as a result of their extreme variety and difficulty to be defined (e.g., are “celery,” “soup,” or “minced meat” materials? What about composite foods such as “moussaka”?). Still, food is a very important class of entities at the boundary between materials and objects, and understanding the neural processing of food has recently been gaining increasing attention (49, 50). We hypothesize that including food stimuli would have substantially increased the number of materials and accordingly introduced additional core dimensions. Future studies should investigate the shared mental representations of objects and materials when also including food, to more fully describe the human mental representational space. For similar reasons, we will need to start considering cross-modal and dynamic representations of objects and materials.

Another potential limitation of our dataset is the chosen scales of material depictions; without an objective criterion for defining close-up photographs, our stimuli might show systematic biases in presenting different materials at different distances, which is known to affect material perception (15). There are also limitations in our current methods of interpreting the discovered dimensions, either by asking human observers or probing a large language model. Even though the labels show that the dimensions capture meaningful and interpretable attributes—which different observers agree upon—words alone are unlikely to be sufficient to capture all the nuances of the perceptual dimensions. It is particularly important, therefore, not to treat the most frequent labels (Fig. 3*E*) as a perfect summary of the dimensions. This would only be problematic if future studies draw on the semantic interpretations alone without considering the actual dimensions, in terms of their values for each image.

One of the most important directions for future research is linking latent spaces defined by material similarity judgments to other behaviors. A key open question is how we flexibly access dimensions of such spaces in a variety of tasks, for example when predicting the likely future behavior of materials in response to external events [e.g., when watching a cloth blowing in the wind; (51)], or planning and executing physical interactions with them [e.g., when poking soft materials with our finger; (52)]. Another key direction for future studies is linking the embedding of material images—and the dimensions relating them to one another—to neural representations. To date, the mapping of materials across cortical regions (53–57) is far less comprehensive than for objects, faces, or places (1, 3, 4, 58–67). A latent space capturing behaviorally relevant dimensions should help us to establish a clearer link between perception and neural activity.

While our work has pointed at a shared representational space between objects and materials and significant overlap in both object and material dimensions, future studies might follow up on this overlap and highlight what material properties are shared with the mental representation of objects and what properties are only relevant when focusing on the comparison of materials. Promising avenues would be to test and model objects and materials simultaneously, and also investigate the overlap in their brain representations.

Finally, future studies should investigate the role of expertise and experience in the representation of materials and material classes. Here, we aimed at identifying the core dimensions that are relevant across many materials and a wide range of naïve observers. However, the mental representation of other observers with different expertise (e.g., botanists) or (visual) experience (e.g., humans living in nonindustrial societies in rainforests) might be significantly different (e.g., with a much more granular representation of organic materials, for example, with more dimensions for distinguishing between plant foliage).

More generally, it would be interesting to ask how these representations are established during development in the first place: To what extent can such embeddings of material images emerge through unsupervised (or weakly supervised) learning processes? Some form of cross-referencing across the senses, as well as active interactions with materials—and perhaps even language—presumably influence perceptual representations of materials, their properties, and classes. However, there is no way for observers to acquire ground truth category labels for most physical properties, so it would be fascinating to test how much and what form of training data is required to learn a latent space resembling human perception.

## Materials and Methods

### Participants.

A total of 7,275 workers from the online crowdsourcing platform Amazon Mechanical Turk participated in the triplet 2-AFC tasks, for the creation of the fully sampled matrix of 60 materials (1,296 workers, 1,238 after exclusion; 638 female, 596 male, 4 other; age was not assessed), for training and evaluating the computational model (5,038 workers, 4,865 after exclusion; 2,798 female, 2,045 male, 22 other; age was not assessed), and for evaluating the separate prediction pipeline on additional images (941 workers, 758 after exclusion; 300 female, 457 male, 1 other; mean age: 36.74 years, SD = 9.37, range = 19 to 81). Workers were excluded if they exhibited overly fast responses (for participants with at least 60 trials, <600 ms response time in >25% of trials or <900 ms response time in >50% of trials) or overly deterministic responses (for participants with at least 160 trials, >60% of responses in one of the 2-AFC positions; expected value, 50%). This removed 18,860 trials for the fully sampled matrix of 60 materials (9.19% of all 205,320 trials), 61,600 trials for the computational model training data (3.3% of all 1,870,700 trials), and 53,020 trials for the 48 out-of-sample materials dataset (25.54% of all 207,560 trials). All workers were located in the United States. All workers provided informed consent and were compensated financially for their time (~6.65 USD/h based on the median response time). In addition, 20 English native speakers (12 female, 8 male; mean age = 34.35, SD = 9.22, range = 25 to 59) took part in the dimension labeling experiment without compensation. All participants provided informed consent. Experimental protocols were approved by the local ethics committee of the Department of Psychology and Sports Sciences of the Justus-Liebig University Giessen (LEK-FB06; application number: 2017-0046) as well as the Ethics Committee of the Medical Faculty of Leipzig (application number: 054/20-ek) and adhered to the declaration of Helsinki.

### STUFF Dataset.

For the selection and identification of material classes in the STUFF dataset, we followed a similar procedure as outlined in (29) for object concepts. Note that the final list is not intended to be a complete and definite set of all picturable material concepts in the English language [see discussion in (29)]. However, the selection procedure yielded a highly systematic and extensive set of picturable material concepts.

Specifically, we based our selection procedure on a list of ~40,000 American English words and two-word expressions, choosing all of these that were tagged as nouns [using part-of-speech tags extended by using the British Lexicon Project; (68, 69)] and achieved a minimum concreteness rating of 4 (level at which the word could be experienced through one of the five basic senses from 1: abstract, to 5: concrete) (70). Next, the resulting 8,669 nouns were screened by 2 authors and 2 student research assistants for whether they reflected materials (i.e., the stuff objects are made from, e.g., granite), with rather liberal inclusion criteria (e.g., also including material composites such as toothpaste), followed by a number of exclusion criteria (*SI Appendix*, *Supplementary Materials and Methods*), leaving us with a list of 200 concrete nouns referring to materials (see *SI Appendix*, Table S1 for a list of all 200 material concepts, together with WordNet keys and definitions). Finally, for each material concept we collected three high-quality close-up naturalistic photographs from the web, showing the material in its typical aggregate state (i.e., at room temperature, e.g., solid iron and liquid oil) and form (e.g., salt grains and concrete walls and floors) without prominently featuring objects. The resulting 600 images of materials were used to collect the similarity judgments (triplet 2-AFC tasks).

### Similarity Judgment (triplet 2-AFC) Task.

To obtain similarities between images under different contexts, we employed an online triplet 2-AFC task that was carried out in sets of 20 trials. All workers were free to choose how many sets they would like to complete. In each trial, we presented three material images in a triangular arrangement on the screen. Participants were told that the image on top was the reference image and that among the two images at the bottom they should choose which one is more similar to that reference. Participants responded with a mouse click, and the next trial started after an intertrial interval of 500 ms. The instructions stated that all images would be showing a type of material or stuff—and if an image would show something they would not call a material, they should base their judgment on their best guess of what the stuff in the image could be. Material triplets and order of presentation were random but chosen in a way that each cell in the 600 × 600 similarity matrix was chosen at least once.

The 60 material images for the creation of the fully sampled matrix were chosen pseudorandomly, in a way that the probability of choosing images from the same material class was as close as possible to the true probability (resulting in 48 unique classes and 6 classes that were chosen twice). The same procedure was applied for the 48 out-of-sample material images, which were chosen from the larger extended set of STUFF materials (38 unique classes, 5 repeated twice).

### Reconstructing the Full Similarity Matrix from the Computational Model.

To derive core dimensions underlying material similarity judgments, we followed a recently developed modeling approach described in more detail in (27, 28) (*SI Appendix*, *Supplementary Materials and Methods*). We defined material similarity in the triplet 2-AFC task as the probability p(*i*,*j*) of the participants choosing material image *i* and *j* to belong together, irrespective of context imposed by image *k* (the third image). Therefore, to compute similarity from the learned embedding for all 600 material images, we obtained the predicted choices from the model for all possible ~107.5 million triplets and then calculated the average choice probability for each pair of material images. The same procedure was used to obtain the fully sampled similarity matrices of 60 and 48 material images, respectively; after obtaining the predicted model choice for all possible triplets, we again calculated the average choice probability for each pair of material images.

### Stability of Modeling Dimensions.

Each time the computational model is trained, the stochasticity of the optimization algorithm might produce a different set of dimensions. To test the stability of the dimensions in our 36-dimensional model, we trained the model 50 times with different random initializations. Then, we correlated each of the 36 original dimensions with all dimensions of one of the 50 reference models and chose the best-fitting dimension across all correlations as the closest match. We averaged the correlations after Fisher z-transformation and then inverted the transformation to get an average reproducibility for each dimension across all 50 models (*SI Appendix*, Fig. S1).

## Supplementary Material

Appendix 01 (PDF)

## Data Availability

Stimuli data have been deposited in OSF (https://osf.io/myutc/) (12). Anonymized Behavioral data and relevant code have been deposited in OSF (https://osf.io/5gr73/) (33).
