# Supplementary material for: Core dimensions of human material perception
Source: Proc Natl Acad Sci U S A. 2025 Mar 5;122(10):e2417202122. doi: 10.1073/pnas.2417202122 (PMC11912425; doi:10.1073/pnas.2417202122)
Supplement: Supplementary file 1 — Appendix 01 (PDF) [file pnas.2417202122.sapp.pdf]

Supporting Information for
Core dimensions of human material perception

Filipp Schmidt[*,†]
Martin N. Hebart[*,†]
Alexandra C. Schmid
Roland W. Fleming

*Corresponding authors: Filipp Schmidt, Martin N. Hebart
† Equal contribution
Email: filipp.schmidt@psychol.uni-giessen.de
martin.hebart@psychiat.med.uni-giessen.de

This PDF file includes:

Supporting text
Figures S1 to S8
Tables S1 to S5
SI References

Supporting text

STUFF dataset criteria. The following procedures were implemented in an iterative procedure where four observers (two naïve and authors F.S. and A.S.) decided on inclusion/exclusion of each word by majority vote. When selecting materials, we excluded all nouns referring to objects (e.g., "coin", "bagpipes"), animals and fictitious creatures (e.g., "cat", "dragon"), people (e.g., "pilot", "father"), navigable places (e.g. "garden"), artwork or crafts (e.g., "collage"), as well as action nouns (e.g. "smack"), times of day ("night"), units and geometric figures (e.g. "quart," "hexagon"), non-visual but sensory nouns (e.g. "click," "music") and nouns that were deemed too difficult to visualize (e.g. "equipment").
Then, we used a number of exclusion criteria to further condense the number of nouns referring to materials: (1) plural form when singular form with the identical meaning is found in the list (e.g. exclude "ashes" when "ash" is present), (2) synonyms (e.g., exclude "chinaware" when "porcelain" is present), (3) invisible materials (e.g., "butane", "gasoline"), with the exception of water, (4) bodily fluids (e.g. "urine"), (5) reference to multiple materials (e.g., "canvas"), (6) drugs and alcohol (e.g., "marijuana", "Irish whiskey"), (7) very rare materials ("plutonium" and "radium"), and (8) food or beverages (e.g., "lemon", "lemonade"). Note that in contrast to [1], we did not choose materials based on whether they were named consistently by participants. Materials are more ambiguous in their appearance compared to objects (e.g., think of "elephant" and "iron"): objects come in a more limited number of typical shapes (and often of typical materials), while materials come in a plethora of shapes. For our purposes, that is, identifying dimensions underlying material similarity judgments, we considered it less important whether participants could name the materials.

Details of computational modelling. To derive core dimensions underlying material similarity judgments, we followed a recently developed modelling described in more detail in [2, 3]. The key concept is a representational embedding, in which material images are characterized as numerical vectors, with each value reflecting a different latent dimension relevant for capturing material similarity judgments. The embedding is initialized with 90 dimensions composed of random numbers, and the model is trained to predict human responses on 90% of triplet responses and tested on the remaining 10%, under the constraints of sparse, continuous and positive dimensions. Please note that the 1,200 repeated triplets that were used to estimate consistency across participants were not included in training or test sets.
The model was implemented in PyTorch 1.6 (https://github.com/ViCCo-Group/SPoSE). Each triplet was encoded using three one-hot vectors (length, 200), and each vector was linked to 90 latent dimensions, but with weights replicated across all three vectors. The 200 $\times$ 90 weights were initialized randomly (range, 0–1). The dot product was chosen as a basis for proximity for computational reasons, but previous results showed similar performance when using the Euclidean distance [3]. The loss function of the model optimization consisted of the cross-entropy, which is the logarithm of the softmax function, and a regularization term based on the L1 norm:

$$\sum^{n} log\left(\frac{exp(x_i x_j)}{exp(x_i x_j) + exp(x_i x_k)}\right) + \lambda \sum^{m} \|x\|_1$$

where x corresponds to an object vector; *i*, *j* and *k* to the indices of the current triplet; *n* to the number of triplets; and *m* to the number of material images. The regularization parameter λ, which controls the trade-off between sparsity and model performance, was determined using cross-validation on the training set (λ = 0.0038). The sparsity constraint results in a penalty for the number of dimensions, so that the resulting model will have fewer dimensions than the 90 initialized dimensions.

The weights in the embedding *X* were enforced to be positive to support interpretability of dimensions. The minimization of the loss was carried out using stochastic gradient descent as implemented in the Adam algorithm [4] using default parameters and a minibatch size of 100 triplets. After the optimization was complete, only dimensions were kept for which at least one material image had a weight larger than 0.1, leaving us with 36 dimensions. The dimensions were sorted in descending order by the sum of their weights across materials.

Dimension labelling experiment. To identify the extent to which the retrieved dimensions were interpretable by human participants, we sent them a pictured survey and asked them to provide labels for 36 so-called "rating scales of material properties". These rating scales for each dimension were created

by binning material images according to their dimension values, with 6 bins for values > 0.3, and a separate bin for all values < 0.3 [3]. Each bin contained a maximum of 10 images, with fewer images for very sparse dimensions. A "rating scale" to the left of the image bins marked three positions along the scale as "high", "low" and "not at all" (**Fig. S8**). Participants were asked to come up with verbal labels for these scales, whereby labels can be descriptive words but also categories. They were also specifically instructed to try to take the full range of materials into account. They should provide at least one and up to as many verbal labels for each scale as they considered reasonable.
The results were corrected for typos and spelling, and we added the term "color" to ambiguous color terms (e.g., cream = cream color) and removed "saturation" for colors (e.g., red saturation = red). Then, we removed redundant words (such as "and", "or", "material") and shortened descriptions, replacing the following word endings: "-like" (e.g., jewel-like = jewel), "-ility" (e.g., flexibility = flexible), "-idity" (e.g., fluidity = fluid), and "-en" (e.g., wooden = wood). Finally, we made all terms singular (e.g., minerals = mineral) and removed synonyms (according to WordNet [5], e.g., aqua color = turquoise, cloth = fabric).

Method for automatically generating semantic feature norm scores. In a first step, we generated lists of binary semantic features for all 200 materials with the large language model GPT-3 [6], closely following an approach described in previous work for objects that was shown to rival results generated by humans [7]. This yielded a list of 11,400 binary semantic features with an occurrence probability for each material. Next, we took this list of semantic features and scaled the probabilities using the well-known term-frequency inverse document frequency (tf-idf) [8]. For later comparability between dimensions, we also scaled them to a sum of 1. To identify important features for each dimension, for a given dimension, we next multiplied the scaled occurrence probability for semantic features with the dimension vector and summed this product across all material classes, yielding a score for how important a given feature is for a given dimension across all materials. Since some features are generally more common than others, we finally took the difference between the feature scores of a given dimension and the mean of all other dimensions. The resulting feature dimension scores are plotted in **Table S5**.

Relationship between object similarity judgments and material similarity judgments. Upon close inspection of dimension labels, many of the identified material dimensions appeared to show a partial correspondence with 66 previously identified core dimensions for objects [3, 9]. For example, some object dimensions were also related to color, such as redness or whiteness, while others referenced material concepts, such as "made of metal" "wood-related". This indicates a shared representational space for objects and materials. To more formally address this apparent correspondence, we conducted the same neural network-based dimension prediction approach as described above [10] but predicted the 66 object dimensions for the 600 material images. This provided us with a 66-dimensional vector for all images, allowing us to compute the correspondence between object and material similarities and the one-to-one mapping of identified dimensions. A comparison of the neural network-based predicted object similarity on material images and the material embedding-predicted similarity matrix yielded a medium to high correlation (r = 0.65), indicating a non-negligible overlap in the mental representations of objects and materials. Next, we inspected both representational embeddings and identified dimensions that corresponded most closely, using a greedy pairwise matching approach without replacement. The average pairwise correlation of dimensions across all 36 dimensions was r = 0.55, with the closest correspondence for "fabric" vs. "textile" (r = 0.87), "green" vs. "green" (r = 0.82), and "small" vs. "coarse-patterned" (r = 0.81), and the smallest correspondence for "blue" vs. "coldness-related" (r = 0.25), "turquoise" vs. "medicine-related", and "cream color" vs. "food-related" (r = 0.15). Together, these results suggest a partial overlap in the representational dimensions of objects and materials, highlighting the role of material properties including colors also for object similarity judgments (see **Fig. S7** for a comparison of all dimension pairs).

Supplementary Figures

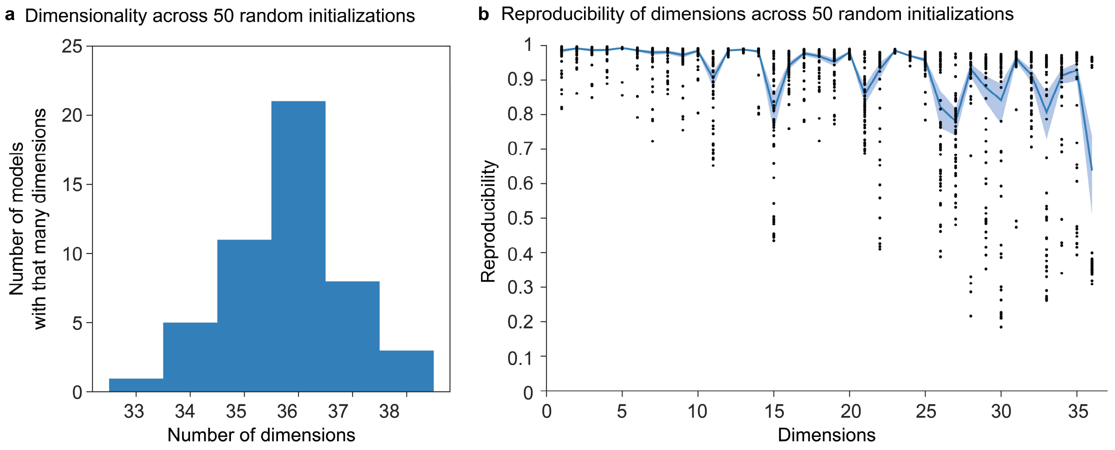

Fig. S1. Stability of model dimensions. A. Dimensionality across 50 random initializations, evaluated by plotting the frequency of resulting models, grouped by their number of dimensions. **B.** Reproducibility of dimensions across 50 random initializations in the chosen 36-dimensional embedding (Pearson's r). Shaded areas are indicating 95% confidence intervals.

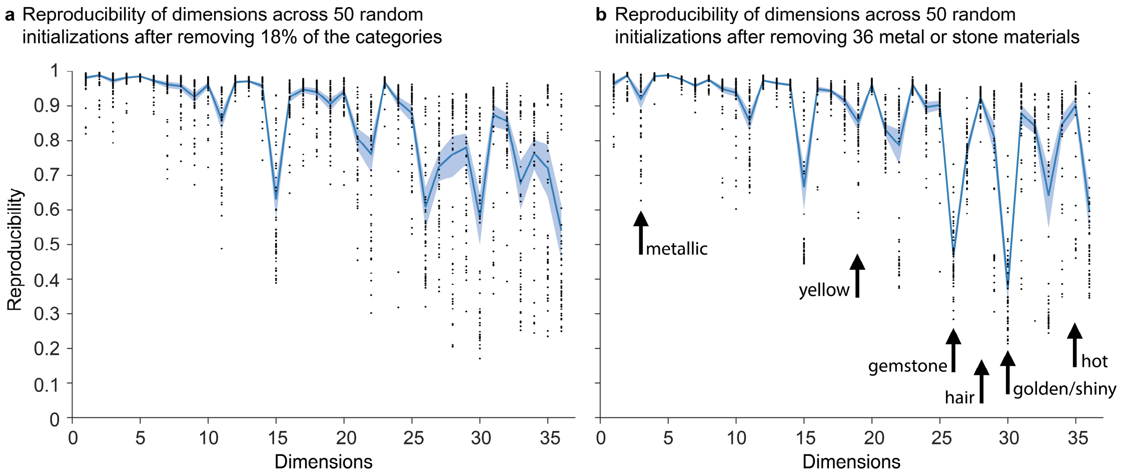

Fig. S2. Reproducibility of model dimensions after removing categories. A. Reproducibility of dimensions across 50 random initializations after randomly removing 18% of the categories in the chosen 36-dimensional embedding (Pearson's r). Shaded areas indicate 95% confidence intervals. B. Reproducibility of dimensions across 50 random initializations after randomly removing 36 metal or stone material categories (corresponding to 18% of the categories) in the chosen 36-dimensional embedding (Pearson's r). Arrows highlight where the two graphs differ, specifically, showing lower reproducibility after removing metal or stone materials for dimensions "metallic" (3), "yellow" (19), "gemstone" (26), "hair" (28), "golden, shiny" (30), and "hot" (35). Again, shaded areas are indicating 95% confidence intervals.

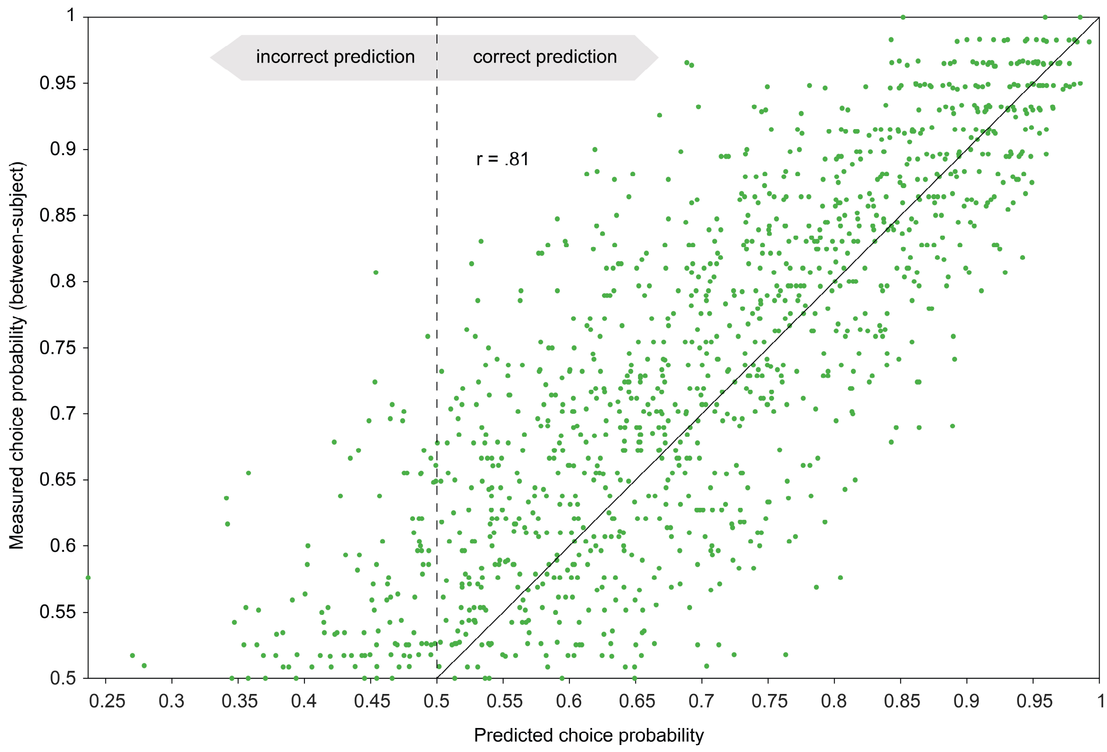

Fig. S3. Model prediction of choice probability. Relationship between the model's predicted choice probability for the 1,200 test triplets and the measured choice probability.

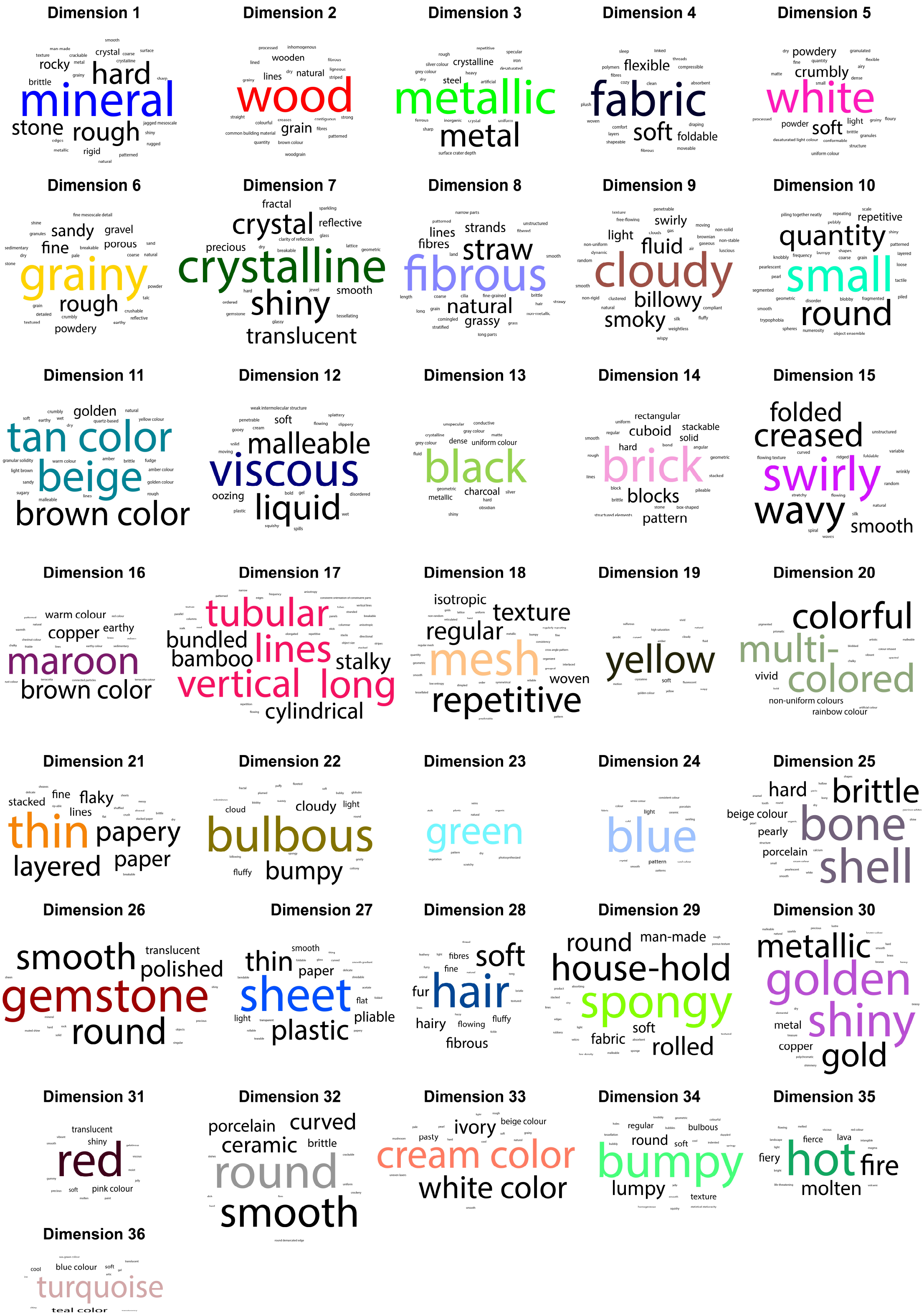

Fig. S4. Word cloud visualizations of the responses obtained in the dimension labelling experiment. Each word cloud shows the semantic labels for one of the 36 dimensions, the size of the words is scaled to the observed frequencies.

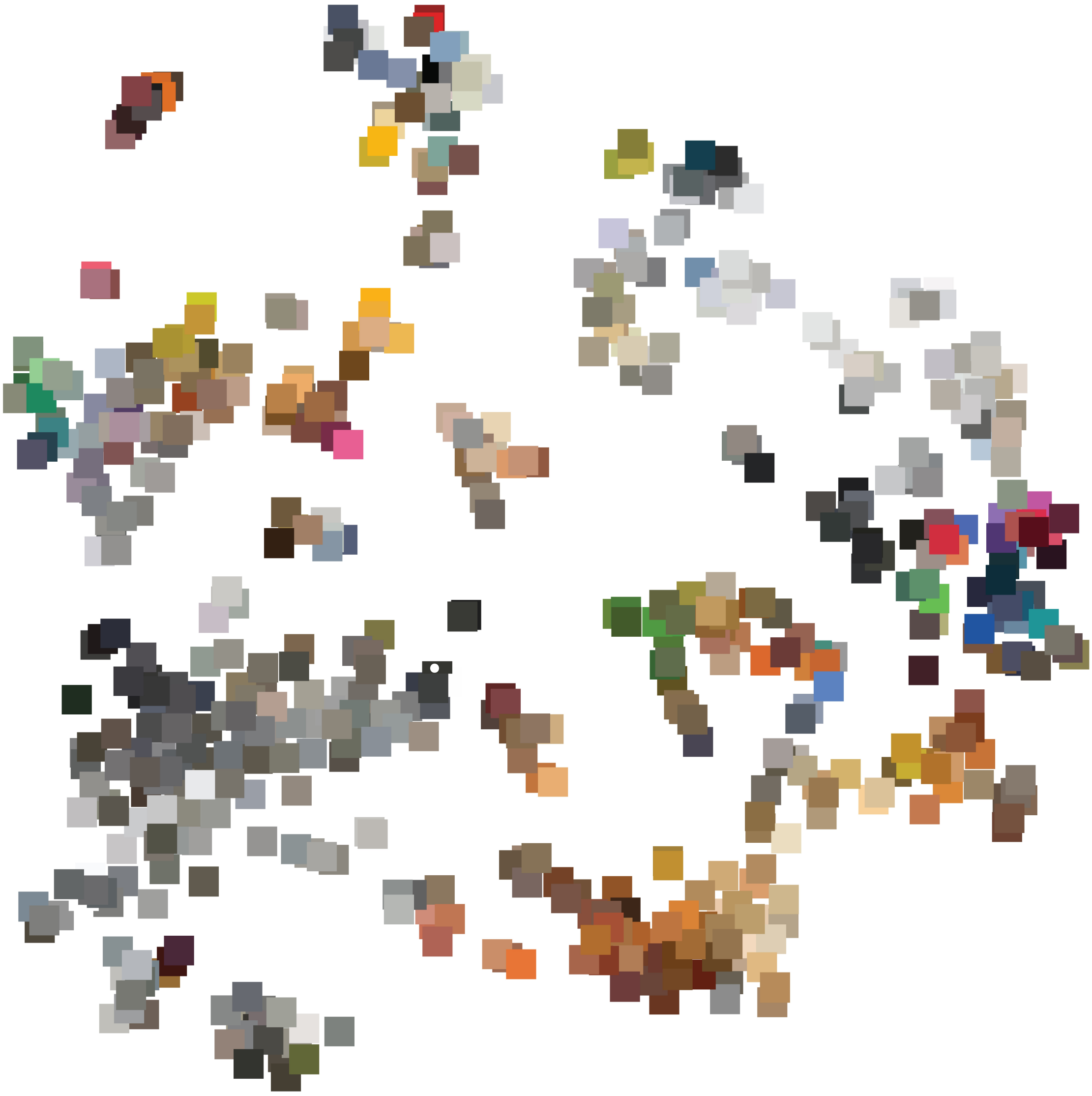

Fig. S5. Two-dimensional visualization of the similarity embedding. The similarity embedding is visualized as described in Fig. 6 but each of the 600 images is represented by a square patch with its median color in CIELAB space, obtained by calculating the median within each L*, a*, and b* axis.

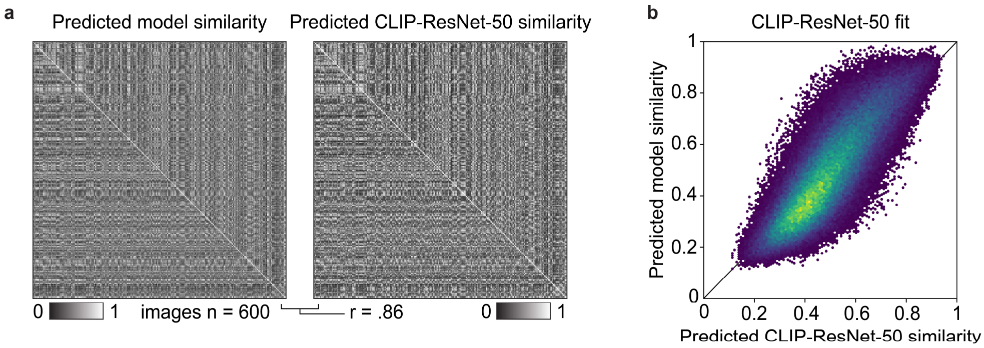

Fig. S6. Prediction by CLIP-ResNet-50. A. To estimate how well the multimodal deep neural network predicted behavioral similarity, we compared the model-generated similarity matrix for all 600 images (left) to the similarity matrix generated from predictions of dimension values from CLIP-ResNet-50 activations (right). B. The good fit between the two shows that we can predict dimension values and similarities of novel images from deep neural network activations.

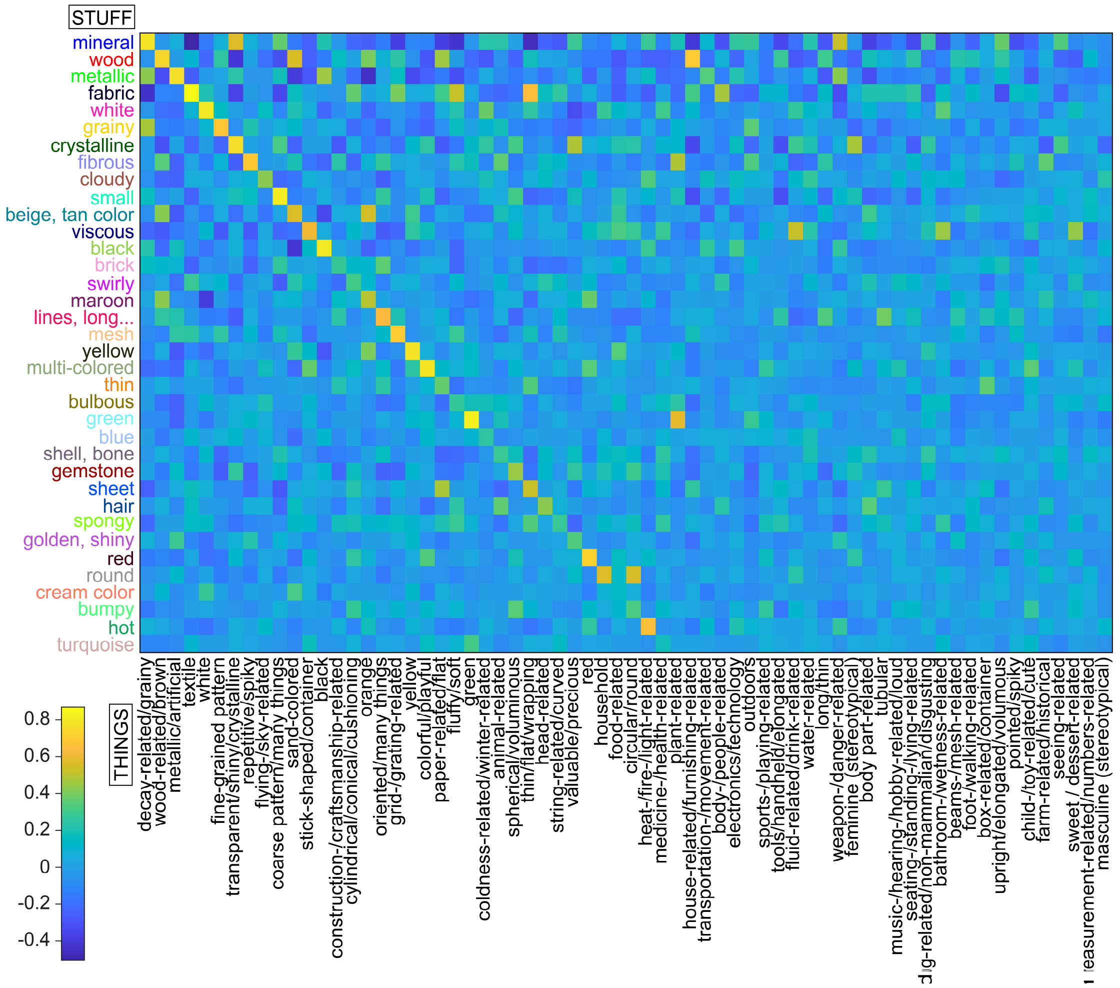

Fig. S7. Correspondence between core dimensions of materials and objects. Plotted are pairwise correlations between dimension values across all 600 material images, with the values for the 66 object dimensions [3] predicted from neural network activations [10].

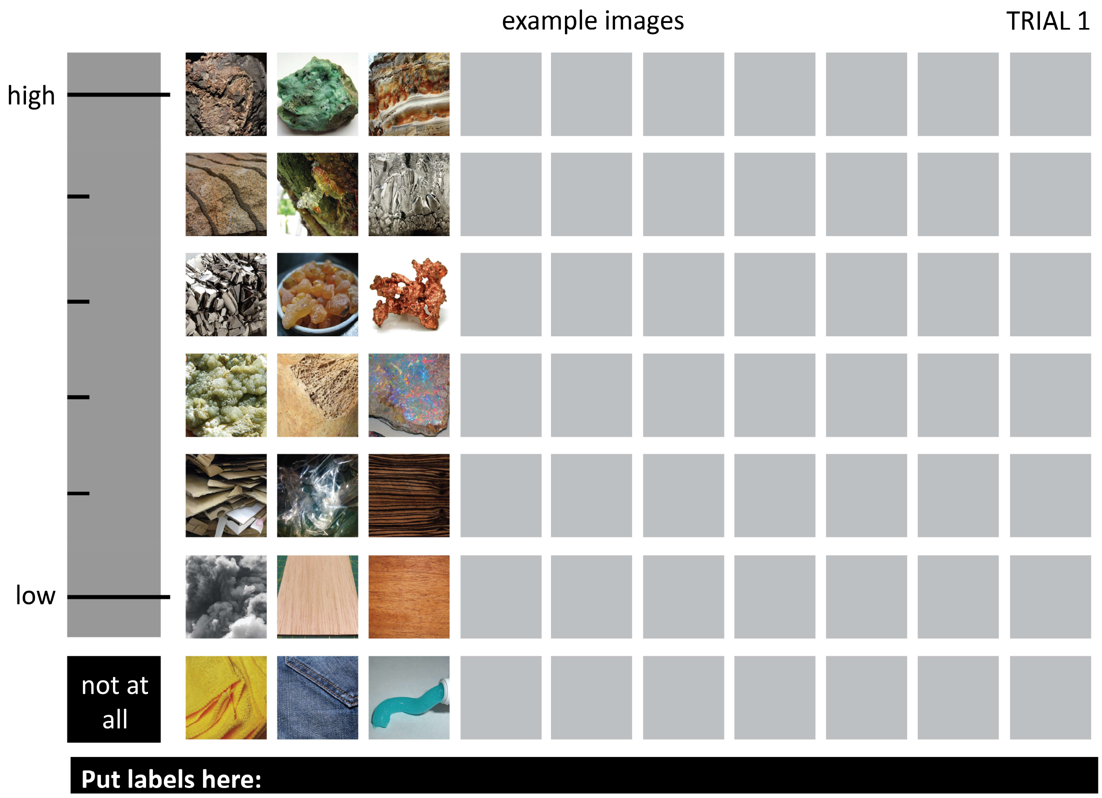

Fig. S8. Example trial in the dimension labelling experiment. As an example, we show the stimulus display for dimension 1, where participants are given examples scoring higher, lower, or not at all on the dimension, and should provide semantic labels. For copyright reasons, we show only three out of ten images in each row. Copyright information for all images is provided in **Table S2**.

Supplementary Tables

Table S1. List of all 200 material concepts, WordNet synset, and definitions. Semantic similarity was calculated by correlating 300-dimensional sense embedding vectors for each of these nouns [11, 12], and, when not available (three classes: play dough, teflon, vaseline), we used the lexical semantic Wu-Palmer similarity measure based on depth of nodes in the WordNet taxonomies [5, 13].

Material class	WordNet Synset	WordNet Definition
algae	algae.n.1	primitive chlorophyll-containing mainly aquatic eukaryotic organisms lacking true stems and roots and leaves
aluminium	aluminium.n.1	a silvery ductile metallic element found primarily in bauxite
amber	amber.n.2	a hard yellowish to brownish translucent fossil resin; used for jewelry
amethyst	amethyst.n.1	a transparent purple variety of quartz; used as a gemstone
arsenic	arsenic.n.2	a very poisonous metallic element that has three allotropic forms; arsenic and arsenic compounds are used as herbicides and insecticides and various alloys; found in arsenopyrite and orpiment and realgar
asbestos	asbestos.n.1	fibrous amphibole; used for making fireproof articles; inhaling fibers can cause asbestosis or lung cancer
ash	ash.n.1	the residue that remains when something is burned
asphalt	asphalt.n.1	mixed asphalt and crushed gravel or sand; used especially for paving but also for roofing
balsawood	balsa.n.1	strong lightweight wood of the balsa tree used especially for floats
bamboo	bamboo.n.1	the hard woody stems of bamboo plants; used in construction and crafts and fishing poles
bark	bark.n.1	tough protective covering of the woody stems and roots of trees and other woody plants
beeswax	beeswax.n.1	a yellow to brown wax secreted by honeybees to build honeycombs
bone	bone.n.1	rigid connective tissue that makes up the skeleton of vertebrates
borax	borax.n.1	an ore of boron consisting of hydrated sodium borate; used as a flux or cleansing agent

brass	brass.n.1	an alloy of copper and zinc
brick	brick.n.1	rectangular block of clay baked by the sun or in a kiln; used as a building or paving material
bronze	bronze.n.1	an alloy of copper and tin and sometimes other elements; also any copper-base alloy containing other elements in place of tin
brownstone	brownstone.n.1	a reddish brown sandstone; used in buildings
bubble wrap	bubble_pack.n.1	packaging in which a product is sealed between a cardboard backing and clear plastic cover
cadmium	cadmium.n.1	a soft bluish-white ductile malleable toxic bivalent metallic element; occurs in association with zinc ores)
calcium	calcium.n.1	a white metallic element that burns with a brilliant light; the fifth most abundant element in the earth's crust; an important component of most plants and animals
carbon	carbon.n.1	an abundant nonmetallic tetravalent element occurring in three allotropic forms: amorphous carbon and graphite and diamond; occurs in all organic compounds
carbon paper	carbon_paper.n.1	a thin paper coated on one side with a dark waxy substance (often containing carbon); used to transfer characters from the original to an under sheet of paper
cashmere	cashmere.n.1	a soft fabric made from the wool of the Cashmere goat
cellophane	cellophane.n.1	a transparent paperlike product that is impervious to moisture and used to wrap candy or cigarettes etc.
cement	cement.n.2	a building material that is a powder made of a mixture of calcined limestone and clay; used with water and sand or gravel to make concrete and mortar
chainmail	chain_mail.n.1	flexible armor made of interlinked metal rings
chalk	chalk.n.1	a soft whitish calcite
chalkstone	chalkstone.n.1	a deposit of urates around a joint or in the external ear; diagnostic of advanced or chronic gout
charcoal	charcoal.n.1	a carbonaceous material obtained by heating wood or other organic matter in the absence of air
cheesecloth	cheesecloth.n.1	a coarse loosely woven cotton gauze; originally used to wrap cheeses

chiffon	chiffon.n.1	a sheer fabric of silk or rayon
chlorine	chlorine.n.1	a common nonmetallic element belonging to the halogens; best known as a heavy yellow irritating toxic gas; used to purify water and as a bleaching agent and disinfectant; occurs naturally only as a salt (as in sea water)
chrome	chrome.n.1	another word for chromium when it is used in dyes or pigments (chromium: a hard brittle multivalent metallic element; resistant to corrosion and tarnishing)
cinder	cinder.n.1	a fragment of incombustible matter left after a wood or coal or charcoal fire
clay	clay.n.1	a very fine-grained soil that is plastic when moist but hard when fired
coal	coal.n.1	fossil fuel consisting of carbonized vegetable matter deposited in the Carboniferous period
cobalt	cobalt.n.1	a hard ferromagnetic silver-white bivalent or trivalent metallic element; a trace element in plant and animal nutrition
cobbleston e	cobblestone.n.1	rectangular paving stone with curved top; once used to make roads
concrete	concrete.n.1	a strong hard building material composed of sand and gravel and cement and water
copper	copper.n.1	a ductile malleable reddish-brown corrosion-resistant diamagnetic metallic element; occurs in various minerals but is the only metal that occurs abundantly in large masses; used as an electrical and thermal conductor
coral	coral.n.2	the hard stony skeleton of a Mediterranean coral that has a delicate red or pink color and is used for jewelry
cord	cord.n.4	a cut pile fabric with vertical ribs; usually made of cotton
cork	cork.n.1	outer bark of the cork oak; used for stoppers for bottles etc.
cotton cloth	cotton.n.2	fabric woven from cotton fibers
cotton wool	cotton_wool.n.1	soft silky fibers from cotton plants in their raw state
crepe paper	crepe_paper.n.1	paper with a crinkled texture; usually colored and used for decorations
denim	denim.n.2	a coarse durable twill-weave cotton fabric

diamond	diamond.n.2	very hard native crystalline carbon valued as a gem
ebony	ebony.n.2	hard dark-colored heartwood of the ebony tree; used in cabinetwork and for piano keys
eggshell	eggshell.n.1	the exterior covering of a bird's egg
ember	ember.n.1	a hot fragment of wood or coal that is left from a fire and is glowing or smoldering
emerald	emerald.n.1	a green transparent form of beryl; highly valued as a gemstone
fat	fat.n.1	a soft greasy substance occurring in organic tissue and consisting of a mixture of lipids (mostly triglycerides)
feather	feather.n.1	the light horny waterproof structure forming the external covering of birds
fiberboard	fiberboard.n.1	wallboard composed of wood chips or shavings bonded together with resin and compressed into rigid sheets
fiberglass	fiberglass.n.1	a covering material made of glass fibers in resins
flame	flame.n.1	the process of combustion of inflammable materials producing heat and light and (often) smoke
flannel	flannel.n.1	a soft light woolen fabric; used for clothing
fleece	fleece.n.3	a soft bulky fabric with deep pile; used chiefly for clothing
flint	flint.n.1	a hard kind of stone; a form of silica more opaque than chalcedony
fluorine	fluorine.n.1	a nonmetallic univalent element belonging to the halogens; usually a yellow irritating toxic flammable gas; a powerful oxidizing agent; recovered from fluorite or cryolite or fluorapatite
foam	foam.n.2	a lightweight material in cellular form; made by introducing gas bubbles during manufacture
fog	fog.n.1	droplets of water vapor suspended in the air near the ground
foliage	foliage.n.1	the main organ of photosynthesis and transpiration in higher plants
frankincense	frankincense.n.1	an aromatic gum resin obtained from various Arabian or East African trees; formerly valued for worship and for embalming and fumigation

froth	froth.n.1	(a mass of small bubbles formed in or on a liquid)
fruitwood	fruitwood.n.1	wood of various fruit trees (as apple or cherry or pear) used especially in cabinetwork
fur	fur.n.1	the dressed hairy coat of a mammal
garnet	garnet.n.1	any of a group of hard glassy minerals (silicates of various metals) used as gemstones and as an abrasive
gauze	gauze.n.2	a net of transparent fabric with a loose open weave
gelatin	gelatin.n.1	a colorless water-soluble glutinous protein obtained from animal tissues such as bone and skin
glass	glass.n.1	a brittle transparent solid with irregular atomic structure
glue	glue.n.1	cement consisting of a sticky substance that is used as an adhesive
gold	gold.n.3	a soft yellow malleable ductile (trivalent and univalent) metallic element; occurs mainly as nuggets in rocks and alluvial deposits; does not react with most chemicals but is attacked by chlorine and aqua regia
granite	granite.n.1	plutonic igneous rock having visibly crystalline texture; generally composed of feldspar and mica and quartz
graphite	graphite.n.1	used as a lubricant and as a moderator in nuclear reactors
grass	grass.n.1	narrow-leaved green herbage: grown as lawns; used as pasture for grazing animals; cut and dried as hay
gunpowder	gunpowder.n.1	a mixture of potassium nitrate, charcoal, and sulfur in a 75:15:10 ratio which is used in gunnery, time fuses, and fireworks
hair	hair.n.1	a covering for the body (or parts of it) consisting of a dense growth of threadlike structures (as on the human head); helps to prevent heat loss)
hay	hay.n.1	grass mowed and cured for use as fodder
horn	horn.n.7	the material (mostly keratin) that covers the horns of ungulates and forms hooves and claws and nails
ice	ice.n.1	water frozen in the solid state
ink	ink.n.1	a liquid used for printing or writing or drawing

iron	iron.n.1	a heavy ductile magnetic metallic element; is silver-white in pure form but readily rusts; used in construction and tools and armament; plays a role in the transport of oxygen by the blood
ivory	ivory.n.1	a hard smooth ivory colored dentine that makes up most of the tusks of elephants and walruses
jade	jade.n.1	a semiprecious gemstone that takes a high polish; is usually green but sometimes whitish; consists of jadeite or nephrite
jelly	jelly.n.3	any substance having the consistency of jelly or gelatin
kevlar	polymer.n.1	a naturally occurring or synthetic compound consisting of large molecules made up of a linked series of repeated simple monomers
latex	latex.n.1	a milky exudate from certain plants that coagulates on exposure to air
lava	lava.n.1	rock that in its molten form (as magma) issues from volcanos; lava is what magma is called when it reaches the surface
lead	lead.n.2	a soft heavy toxic malleable metallic element; bluish white when freshly cut but tarnishes readily to dull grey
leather	leather.n.1	an animal skin made smooth and flexible by removing the hair and then tanning
limestone	limestone.n.1	a sedimentary rock consisting mainly of calcium that was deposited by the remains of marine animals
linen	linen.n.1	a fabric woven with fibers from the flax plant
linoleum	linoleum.n.1	a floor covering
magnesium	magnesium.n.1	a light silver-white ductile bivalent metallic element; in pure form it burns with brilliant white flame; occurs naturally only in combination (as in magnesite and dolomite and carnallite and spinel and olivine)
mahogany	mahogany.n.1	wood of any of various mahogany trees; much used for cabinetwork and furniture
marble	marble.n.1	a hard crystalline metamorphic rock that takes a high polish; used for sculpture and as building material
marblewood	marblewood.n.1	hard marbled wood

microfiber	polyester.n.3	any of a large class of synthetic fabrics
moonstone	moonstone.n.1	a transparent or translucent gemstone with a pearly luster; some specimens are orthoclase feldspar and others are plagioclase feldspar
mortar	mortar.n.2	used as a bond in masonry or for covering a wall
moss	moss.n.1	tiny leafy-stemmed flowerless plants
mud	mud.n.1	water soaked soil; soft wet earth
nickel	nickel.n.1	a hard malleable ductile silvery metallic element that is resistant to corrosion; used in alloys; occurs in pentlandite and smaltite and garnierite and millerite
nylon	nylon.n.1	a thermoplastic polyamide; a family of strong resilient synthetic fibers
obsidian	obsidian.n.1	acid or granitic glass formed by the rapid cooling of lava without crystallization; usually dark, but transparent in thin pieces
oil	oil.n.1	a slippery or viscous liquid or liquefiable substance not miscible with water
oilcloth	oilcloth.n.1	cloth treated on one side with a drying oil or synthetic resin
oilpaper	oilpaper.n.1	paper that has been made translucent and waterproof by soaking in oil
oilskin	oilskin.n.1	a macintosh made from cotton fabric treated with oil and pigment to make it waterproof
onionskin	onionskin.n.1	a thin strong lightweight translucent paper used especially for making carbon copies
onyx	onyx.n.1	a chalcedony with alternating black and white bands; used in making cameos
opal	opal.n.1	a translucent mineral consisting of hydrated silica of variable color; some varieties are used as gemstones
paint	paint.n.1	a substance used as a coating to protect or decorate a surface (especially a mixture of pigment suspended in a liquid); dries to form a hard coating
paper	paper.n.1	a material made of cellulose pulp derived mainly from wood or rags or certain grasses

paperboard	paperboard.n.1	a cardboard suitable for making posters
papyrus	papyrus.n.1	paper made from the papyrus plant by cutting it in strips and pressing it flat; used by ancient Egyptians and Greeks and Romans
parchment	parchment.n.1	a superior paper resembling sheepskin; skin of a sheep or goat prepared for writing on
pearl	pearl.n.1	a smooth lustrous round structure inside the shell of a clam or oyster; much valued as a jewel
petroleum	petroleum.n.1	a dark oil consisting mainly of hydrocarbons
pewter	pewter.n.1	any of various alloys of tin with small amounts of other metals (especially lead)
phosphorus	phosphorus.n.1	a multivalent nonmetallic element of the nitrogen family that occurs commonly in inorganic phosphate rocks and as organic phosphates in all living cells; is highly reactive and occurs in several allotropic forms
pinewood	pinewood, pine.n.2	straight-grained durable and often resinous white to yellowish timber of any of numerous trees of the genus Pinus
plaster	plaster.n.1	a mixture of lime or gypsum with sand and water; hardens into a smooth solid; used to cover walls and ceilings
plasterboard	plasterwork.n.1	a surface of hardened plaster (as on a wall or ceiling)
plastic	plastic.n.1	generic name for certain synthetic or semisynthetic materials that can be molded or extruded into objects or films or filaments or used for making e.g. coatings and adhesives
platinum	platinum.n.1	a heavy precious metallic element; grey-white and resistant to corroding; occurs in some nickel and copper ores and is also found native in some deposits
play dough	plasticine.n.1	a synthetic material resembling clay but remaining soft; used as a substitute for clay or wax in modeling (especially in schools)
plywood	plywood.n.1	a laminate made of thin layers of wood
polystyrene	polystyrene.n.1	a polymer of styrene; a rigid transparent thermoplastic
porcelain	porcelain.n.1	ceramic ware made of a more or less translucent ceramic
pottery	pottery.n.1	ceramic ware made from clay and baked in a kiln

quartz	quartz.n.2	a hard glossy mineral consisting of silicon dioxide in crystal form; present in most rocks (especially sandstone and granite); yellow sand is quartz with iron oxide impurities
quartzite	quartzite.n.1	hard metamorphic rock consisting essentially of interlocking quartz crystals
rayon	rayon.n.1	a synthetic silklike fabric
redwood	redwood.n.1	the soft reddish wood of either of two species of sequoia trees
resin	resin.n.1	any of a class of solid or semisolid viscous substances obtained either as exudations from certain plants or prepared by polymerization of simple molecules
rhinestone	rhinestone.n.1	an imitation diamond made from rock crystal or glass or paste
ricepaper	rice_paper.n.1	a thin delicate material resembling paper; made from the rice-paper tree
rosewood	rosewood.n.1	hard dark reddish wood of a rosewood tree having a strongly marked grain; used in cabinetwork
rubber	rubber.n.1	an elastic material obtained from the latex sap of trees (especially trees of the genera Hevea and Ficus) that can be vulcanized and finished into a variety of products
ruby	ruby.n.1	a transparent piece of ruby that has been cut and polished and is valued as a precious gem
salt	salt.n.2	white crystalline form of especially sodium chloride used to season and preserve food
sand	sand.n.1	a loose material consisting of grains of rock or coral
sandalwood	sandalwood.n.1	close-grained fragrant yellowish heartwood of the true sandalwood; has insect repelling properties and is used for carving and cabinetwork
sandpaper	sandpaper.n.1	stiff paper coated with powdered emery or sand
sandstone	sandstone.n.1	a sedimentary rock consisting of sand consolidated with some cement (clay or quartz etc.)
sapphire	sapphire.n.1	a precious transparent stone of rich blue corundum valued as a gemstone

satin	satin.n.1	a smooth fabric of silk or rayon; has a glossy face and a dull back
satinwood	satinwood.n.2	hard yellowish wood of a satinwood tree having a satiny luster; used for fine cabinetwork and tools
sequin	sequin.n.1	adornment consisting of a small piece of shiny material used to decorate clothing
shell	shell.n.3	hard outer covering or case of certain organisms such as arthropods and turtles
silicon	silicon.n.1	a tetravalent nonmetallic element; next to oxygen it is the most abundant element in the earth's crust; occurs in clay and feldspar and granite and quartz and sand; used as a semiconductor in transistors
silicone	silicone.n.1	any of a large class of siloxanes that are unusually stable over a wide range of temperatures; used in lubricants and adhesives and coatings and synthetic rubber and electrical insulation
silk	silk.n.1	a fabric made from the fine threads produced by certain insect larvae
silver	silver.n.1	a soft white precious univalent metallic element having the highest electrical and thermal conductivity of any metal; occurs in argentite and in free form; used in coins and jewelry and tableware and photography
skin	skin.n.1	a natural protective body covering and site of the sense of touch; body covering of a living animal
slate	slate.n.3	a fine-grained metamorphic rock that can be split into thin layers
smoke	smoke.n.1	a cloud of fine particles suspended in a gas
snow	snow.n.1	precipitation falling from clouds in the form of ice crystals
soap	soap.n.1	a cleansing agent made from the salts of vegetable or animal fats
soil	soil.n.3	material in the top layer of the surface of the earth in which plants can grow (especially with reference to its quality or use)
spandex	spandex.n.1	an elastic synthetic fabric
sponge	sponge.n.1	a porous mass of interlacing fibers that forms the internal skeleton of various marine animals and usable to absorb

		water or any porous rubber or cellulose product similarly used
steel	steel.n.1	an alloy of iron with small amounts of carbon; widely used in construction; mechanical properties can be varied over a wide range
straw	straw.n.2	material consisting of seed coverings and small pieces of stem or leaves that have been separated from the seeds
suede	suede.n.1	leather with a napped surface
sugar	sugar.n.1	a white crystalline carbohydrate used as a sweetener and preservative
sulfur	sulfur.n.1	an abundant tasteless odorless multivalent nonmetallic element; best known in yellow crystals; occurs in many sulphide and sulphate minerals and even in native form (especially in volcanic regions)
talc	talc.n.1	a fine grained mineral having a soft soapy feel and consisting of hydrated magnesium silicate; used in a variety of products including talcum powder
talcum powder	talcum_powder.n.1	a toilet powder made of purified talc and usually scented; absorbs excess moisture
tar	tar.n.1	any of various dark heavy viscid substances obtained as a residue
tarpaper	tar_paper.n.1	a heavy paper impregnated with tar and used as part of a roof for waterproofing
teakwood	teakwood.n.1	hard strong durable yellowish-brown wood of teak trees; resistant to insects and to warping; used for furniture and in shipbuilding
teflon	teflon.n.1	a material used to coat cooking utensils and in industrial applications where sticking is to be avoided
terracotta	terra_cotta.n.1	a hard unglazed brownish-red earthenware
tin	tin.n.1	a silvery malleable metallic element that resists corrosion; used in many alloys and to coat other metals to prevent corrosion; obtained chiefly from cassiterite where it occurs as tin oxide
tinfoil	tinfoil.n.1	foil made of tin or an alloy of tin and lead
tinsel	tinsel.n.2	a thread with glittering metal foil attached

titanium	titanium.n.1	a light strong grey lustrous corrosion-resistant metallic element used in strong lightweight alloys (as for airplane parts); the main sources are rutile and ilmenite
toilet paper	toilet_paper.n.1	a soft thin absorbent paper for use in toilets
tooth	tooth.n.1	hard bonelike structures in the jaws of vertebrates; used for biting and chewing or for attack and defense
toothpaste	toothpaste.n.1	a dentifrice in the form of a paste
topaz	topaz.n.2	a mineral (fluosilicate of aluminum) that occurs in crystals of various colors and is used as a gemstone
tungsten	tungsten.n.1	a heavy grey-white metallic element; the pure form is used mainly in electrical applications; it is found in several ores including wolframite and scheelite
tweed	tweed.n.1	thick woolen fabric used for clothing; originated in Scotland
uranium	uranium.n.1	a heavy toxic silvery-white radioactive metallic element; occurs in many isotopes; used for nuclear fuels and nuclear weapons
vapor	vapor.n.1	a visible suspension in the air of particles of some substance
vaseline	vaseline.n.1	a trademarked brand of petroleum jelly
velcro	velcro.n.1	nylon fabric used as a fastening
velvet	velvet.n.1	a silky densely piled fabric with a plain back
vinyl	vinyl.n.2	shiny and tough and flexible plastic; used especially for floor coverings
water	water.n.1	binary compound that occurs at room temperature as a clear colorless odorless tasteless liquid; freezes into ice below 0 degrees centigrade and boils above 100 degrees centigrade; widely used as a solvent
wax	wax.n.1	any of various substances of either mineral origin or plant or animal origin; they are solid at normal temperatures and insoluble in water
wax paper	wax_paper.n.1	paper that has been waterproofed by treatment with wax or paraffin
wool	wool.n.1	a fabric made from the hair of sheep

zinc	zinc.n.1	a bluish-white lustrous metallic element; brittle at ordinary temperatures but malleable when heated; used in a wide variety of alloys and in galvanizing iron; it occurs naturally as zinc sulphide in zinc blende
zirconium	zirconium.n.1	a lustrous grey strong metallic element resembling titanium; it is used in nuclear reactors as a neutron absorber; it occurs in baddeleyite but is obtained chiefly from zircon

Table S2. List of copyright information for all material images reprinted in this paper.

material class	Creator	Platform	Licence
aluminium	myfreetextures.com	myfreetextures.com	MyFreeTextures License
arsenic	Parent Géry	wikimedia.org	CC0 1.0
ash	Laurentius	wikimedia.org	CC BY-SA 3.0
asphalt	Kunzinger	flickr.com	CC BY 2.0
balsawood	mauro halpern	flickr.com	CC BY 2.0
balsawood	Joseph Francis	flickr.com	CC BY 2.0
beeswax	Maja Dumat	flickr.com	CC BY 2.0
bone	Maky Orel	wikimedia.org	CC0 1.0
borax	Rock Currier	wikimedia.org	CC BY 3.0
borax	Rock Currier	wikimedia.org	CC BY 3.0
borax	Ra'ike	wikimedia.org	CC BY-SA 3.0
brownstone	Richter.V	flickr.com	CC BY-NC-SA 2.0
cellophane	nsmithtnz	flickr.com	CC BY-NC 2.0
cellophane	Itzuvit	wikimedia.org	CC BY-SA 3.0
cement	Marcu Ioachim	flickr.com	CC0 1.0
cement	Marcu Ioachim	flickr.com	CC0 1.0
chalkstone	su-lin	flickr.com	CC BY-NC-ND 2.0
chrome	Alchemist-hp	wikimedia.org	CC BY-NC-ND 3.0
clay	pixabay	pexels.com	CC0
cobalt	James St. John	wikimedia.org	CC BY 2.0
cobalt	Alchemist-hp	wikimedia.org	CC BY-NC-ND 3.0
concrete	Philippe Ramakers	unsplash.com	Unsplash Licence, https://unsplash.com/license

copper	Jonathan Zander	wikimedia.org	CC BY-SA 2.5
copper	Joanna Kosinska	unsplash.com	Unsplash Licence, https://unsplash.com/license
cork	Grapebowl	wikimedia.org	CC BY-SA 2.5
cotton wool	Ross Elliott	flickr.com	CC BY 2.0
crepe paper	under the same moon	flickr.com	CC BY 2.0
denim	Creativity103	flickr.com	CC BY 2.0
denim	Uwe_Jelting	pixabay.com	Pixabay License; https://pixabay.com/en/service/license/
ebony	Makassar-Ebenholz	wikimedia.org	CC BY-SA 2.5
ember	Heili Rüütel	flickr.com	Personal reprint permission
fat	Nikol Lohr	flickr.com	CC BY-NC-SA 2.0
fiberglass	Cjp24	wikimedia.org	CC BY-SA 3.0
flannel	Sean Ganann	flickr.com	CC BY-NC-SA 2.0
foam	Windell Oskay	flickr.com	CC BY-SA 2.0
foam	Дар Ветер	wikimedia.org	CC BY-SA 3.0
foliage	Anne Worner	flickr.com	CC BY-NC-SA 2.0
foliage	Timothy Clough	flickr.com	CC BY-NC-SA 2.0
frankincense	Lupus in Saxonia	wikimedia.org	CC BY-SA 4.0
froth	Stefan Knaake	flickr.com	CC BY-NC 2.0
fur	Sheila in Moonducks	flickr.com	CC BY 2.0
garnet	Rachael Moore	flickr.com	CC BY-NC 2.0
glass	makamuki0	pixabay.com	Pixabay License, https://pixabay.com/en/service/license/

graphite	James St. John	flickr.com	CC BY 2.0
graphite	Ra'ike	wikimedia.org	CC BY-SA 3.0
horn	Zachi Evenor	wikimedia.org	CC BY-SA 4.0
iron	michael schaffler	unsplash.com	Unsplash Licence, https://unsplash.com/license
jade	Ra'ike	wikimedia.org	CC BY 3.0
leather	Cinnamon Cooper	flickr.com	CC BY-NC-SA 2.0
limestone	Manishwiki15	wikimedia.org	CC BY-SA 3.0
linen	Sincerely Media	unsplash.com	Unsplash Licence, https://unsplash.com/license
magnesium	Mark Fergus	wikimedia.org	CC BY 3.0
mahogany	Das Ohr	wikimedia.org	CC BY-SA 3.0
marble	shaireproductions.co m	flickr.com	CC BY 2.0
marble	Olga V	pexels.com	Public domain
microfiber	Chris Yarzab	flickr.com	CC BY-NC-SA 2.0
moonstone	Robert M. Lavinsky	wikimedia.org	CC BY-SA 3.0
moonstone	Jarno	wikimedia.org	CC BY 2.0
moonstone	Géry PARENT	wikimedia.org	CC0 1.0
obsidian	Ji-Elle	wikimedia.org	CC BY-SA 3.0
oil	Martin Cooper	flickr.com	CC BY 2.0
opal	James St. John	wikimedia.org	CC-BY-2.0
paper	Niklas Bildhauer	wikimedia.org	CC BY-SA 2.0
paperboard	boris drenec	flickr.com	CC BY-NC-SA 2.0
phosphorus	Dnn87	wikimedia.org	CC BY 3.0
pinewood	decar66	flickr.com	CC BY-NC-SA 2.0

plaster	tropicalart77 (Tammy Dial Gray)	flickr.com	CC BY 2.0
plaster	Lara604	flickr.com	CC BY 2.0
plastic	freeimages.co.uk	freeimages.co.uk	Freeimage License
platinum	Alchemist-hp	wikimedia.org	CC BY-NC-ND 3.0
quartz	Andrew Gustar	flickr.com	CC BY-ND 2.0
resin	Darkone	wikimedia.org	CC BY-SA 2.5
resin	Emmanuel Boutet	wikimedia.org	CC BY-SA 3.0
rubber	bgfons.com	bgfons.com	CC BY-NC 4.0
salt	Gerwin Sturm	flickr.com	CC BY-SA 2.0
salt	Christian Mertes	wikimedia.org	CC BY-SA 3.0
sandstone	Kasia Trapszo	flickr.com	CC BY-NC-SA 2.0
satin	Jwrusa & Kjoonlee	wikimedia.org	CC0 1.0
satinwood	Philiipp Zinger	wikimedia.org	CC BY-SA 4.0
silicon	Enricoros	wikimedia.org	CC0 1.0
silver	Roger Culos	wikimedia.org	CC BY-SA 4.0
smoke	pxhere.com	pxhere.com	CC0 1.0
smoke	Jens Johnsson	unsplash.com	Unsplash Licence,
soil	Elvis Ripley	flickr.com	https://unsplash.com/license
soil	United Soybean Board	flickr.com	CC BY-NC 2.0
sugar	Marco Verch Professional Photographer	flickr.com	CC BY 2.0
sulfur	Parent Géry	wikimedia.org	CC BY 2.0
talc	Stephanie Clifford	flickr.com	CC0 1.0

talc	John Krygier	wikimedia.org	CC BY 2.0
tar	ariari	flickr.com	CC0 1.0
tar	Linda, Fortuna future	flickr.com	CC BY-NC-ND 2.0
teflon	Fructibus	wikimedia.org	CC BY-NC 2.0
tin	Schtone	wikimedia.org	CC0
tin	Attribution not necessary	pexels.com	CC0 1.0
titanium	Alchemist-hp	wikimedia.org	Pexels License
toothpaste	SCEhardt	wikimedia.org	CC BY-NC-ND 3.0
toothpaste	Photos8	wikimedia.org	CC0 1.0
topaz	Photos8	wikimedia.org	CC BY 3.0
tungsten	Alchemist-hp	wikimedia.org	CC BY-SA 4.0
tweed	PKM	wikimedia.org	CC BY-NC-ND 3.0
vaseline	Kiyok	wikimedia.org	CC0 1.0
velvet	Steve Miller	flickr.com	CC BY-SA 3.0
velvet	Kristiana Pinne	unsplash.com	Unsplash Licence, https://unsplash.com/license
water	Artiom Vallat	unsplash.com	Unsplash Licence, https://unsplash.com/license
water	stux	pixabay.com	Pixabay License, https://pixabay.com/en/service/license/

Table S3. List of semantic labels in the dimension labeling task with at least 20% agreement between participants.

dimension	Labels (frequencies)
1	mineral (11), hard (7), rough (6), stone (5)
2	wood (10)
3	metallic (10), metal (7)
4	fabric (14), soft (7), flexible (4),
5	white (12), soft (5), crumbly (4),
6	grainy (8), rough (4)
7	crystalline (8), shiny (6), crystal (5), translucent (4)
8	fibrous (6), straw (4)
9	cloudy (6)
10	small (8), round (6), quantity (5)
11	beige (5), tan color (5), brown (4)
12	viscous (8), liquid (6), malleable (5)
13	black (16)
14	brick (10), blocks (4)
15	swirly (7), wavy (6), creased (5), folded (4)
16	maroon (6), brown (4)
17	lines (3), long (3), tubular (3), vertical (3)
18	mesh (5), repetitive (4)
19	yellow (12)
20	multi-colored (9), colorful (8)
21	thin (8), layered (5), papery (5), paper (4)
22	bulbous (9), bumpy (5)

23	green (16)
24	blue (17)
25	bone (7), shell (7), brittle (5)
26	gemstone (8), round (6), smooth (6), polished (4)
27	sheet (10), plastic (6), thin (6)
28	hair (9), soft (6)
29	spongy (5), house-hold (4)
30	golden (7), shiny (7), gold (5), metallic (5)
31	red (17)
32	round (8), smooth (7), ceramic (4), curved (4)
33	cream color (8), white (6), ivory (4)
34	bumpy (9), lumpy (4)
35	hot (12), fire (7), molten (5)
36	turquoise (12), teal (6), blue (4)

Table S4. Three most frequent descriptive labels for all 200 material concepts obtained from GPT-3 feature norms.

**Material class**			
algae	is green	a plant	is slimy
aluminium	is shiny	a metal	is light
amber	is yellow	used for jewelry	a fossil
amethyst	is purple	a gemstone	is transparent
arsenic	is poisonous	a metal	is white
asbestos	a mineral	is white	found in rocks
ash	is grey	made of wood	has leaves
asphalt	is black	is sticky	used for roads
balsawood	is light	made of wood	is hard
bamboo	has leaves	a plant	used for making furniture
bark	is brown	is rough	is hard
beeswax	is yellow	made by bees	used for candles
bone	is hard	is white	has marrow
borax	is white	used for cleaning	a chemical
brass	is yellow	is shiny	a metal
brick	made of clay	used for building houses	is red
bronze	a metal	is shiny	used for statues
brownstone	used for buildings	a type of rock	made of stone
bubble wrap	made of plastic	has bubbles	used for shipping
cadmium	a metal	is toxic	used in batteries
calcium	a mineral	is white	found in milk
carbon	is black	found in coal	found in diamonds

carbon paper	used for writing	used for copying	is thin
cashmere	is soft	is expensive	is warm
cellophane	is thin	is clear	is transparent
cement	is hard	is strong	is grey
chainmail	made of metal	worn by knights	is heavy
chalk	is white	used for writing	used for writing on blackboards
chalkstone	is white	is soft	a rock
charcoal	is black	used for cooking	made from wood
cheesecloth	is white	made of cotton	used for straining
chiffon	made of silk	is transparent	is thin
chlorine	is poisonous	a gas	a chemical
chrome	is shiny	a metal	is hard
cinder	is black	a rock	is hot
clay	used for making pots	used for making pottery	used for pottery
coal	is black	used for heating	found in mines
cobalt	a metal	is blue	used in batteries
cobblestone	made of stone	is hard	used for paving roads
concrete	is strong	is hard	used for roads
copper	a metal	is shiny	conducts electricity
coral	is red	found in the ocean	is pink
cord	is strong	made of rubber	made of plastic
cork	comes from trees	floats	floats on water
cotton cloth	is white	is soft	made of cotton

cotton wool	is soft	is white	used for cleaning
crepe paper	is thin	used for wrapping gifts	made of paper
denim	is blue	a fabric	made of cotton
diamond	is hard	is expensive	is clear
ebony	is black	is hard	a wood
eggshell	is white	is hard	made of calcium
ember	is hot	is red	used for cooking
emerald	is green	is precious	a gemstone
fat	is white	used for cooking	used in cooking
feather	is light	is soft	has a quill
fiberboard	made of wood	is strong	has a smooth surface
fiberglass	is strong	used for insulation	used in boats
flame	is hot	burns	is red
flannel	is soft	made of cotton	is warm
fleece	is warm	is soft	used for clothing
flint	is hard	a rock	used to make fire
fluorine	a gas	is poisonous	used in toothpaste
foam	is white	has bubbles	is soft
fog	is white	is cold	is wet
foliage	is green	is on trees	has leaves
frankincense	comes from trees	used in religious ceremonies	used in churches
froth	is foamy	is white	has bubbles
fruitwood	is hard	used for carving	used for furniture

fur	is soft	used for clothing	is warm
garnet	is red	a gemstone	a stone
gauze	is white	is thin	used for bandages
gelatin	a food	is clear	a dessert
glass	is transparent	is brittle	is hard
glue	is sticky	is white	used for sticking things together
gold	is yellow	is shiny	a metal
granite	is hard	a rock	is grey
graphite	is soft	is black	a mineral
grass	is green	has seeds	a plant
gunpowder	is explosive	is black	used in fireworks
hair	made of keratin	is long	made of protein
hay	used for feeding animals	is brown	made of grass
horn	is hard	made of bone	made of metal
ice	is cold	is frozen	is frozen water
ink	is black	used for writing	used for printing
iron	is strong	a metal	is heavy
ivory	is white	is hard	comes from elephants
jade	is green	a gemstone	a stone
jelly	is sweet	made of sugar	a dessert
kevlar	is strong	used in bulletproof vests	used for protection
latex	is sticky	is white	used in balloons
lava	is hot	is red	flows

lead	is heavy	a metal	is soft
leather	used to make belts	used to make shoes	used to make wallets
limestone	a rock	used for building	found in caves
linen	is white	is soft	made of flax
linoleum	made of plastic	used for floors	has a pattern
magnesium	a metal	is shiny	is white
mahogany	is hard	used for furniture	a wood
marble	is white	is smooth	is shiny
marblewood	is hard	used for making furniture	used for furniture
microfiber	used for cleaning	made of plastic	is soft
moonstone	is white	a gemstone	is shiny
mortar	used for construction	made of cement	has a handle
moss	is green	grows on trees	a plant
mud	is sticky	is brown	is dirty
nickel	a metal	is shiny	is hard
nylon	is strong	a fabric	used for stockings
obsidian	is black	is shiny	is sharp
oil	used for cooking	is black	is thick
oilcloth	is waterproof	made of plastic	used for covering tables
oilpaper	used for wrapping food	is thin	made of paper
oilskin	is waterproof	used for clothing	has a hood
onionskin	is thin	is white	used for writing
onyx	is black	is shiny	a gemstone

opal	a gemstone	is precious	a stone
paint	used for painting	used for walls	has a smell
paper	is white	used for writing	is thin
paperboard	used for packaging	is white	is thin
papyrus	used for writing	a plant	has leaves
parchment	used for writing	made of animal skin	is thin
pearl	is white	is shiny	is round
petroleum	a liquid	used for fuel	is black
pewter	is shiny	made of metal	a metal
phosphorus	is a solid	a chemical element	is white
pinewood	used for making furniture	used for making houses	used for building
plaster	is white	used to make casts	used for bandages
plasterboard	is white	a building material	has a smooth surface
plastic	made of oil	used for packaging	is hard
platinum	a metal	is shiny	is expensive
play dough	is soft	made of flour	used for play
plywood	made of wood	is strong	used for making furniture
polystyrene	is white	used for packaging	made of plastic
porcelain	is white	made of clay	is fragile
pottery	made of clay	has a handle	has a lid
quartz	is hard	is clear	a mineral
quartzite	is hard	a rock	used for building
rayon	made from cotton	made from wood	made from bamboo

redwood	a tree	has branches	is tall
resin	is sticky	comes from trees	is hard
rhinestone	is shiny	made of glass	used for decoration
rice paper	is thin	is white	made of rice
rosewood	is hard	used for furniture	used for making musical instruments
rubber	is elastic	is sticky	is black
ruby	is red	a stone	is precious
salt	is white	a mineral	used in cooking
sand	is white	found on beaches	found in deserts
sandalwood	a tree	used for making furniture	used in incense
sandpaper	is rough	made of paper	used for sanding
sandstone	has grains	made of sand	a rock
sapphire	is blue	a gemstone	a stone
satin	is shiny	is smooth	a fabric
satinwood	a wood	used for furniture	is hard
sequin	is shiny	used for decoration	made of metal
shell	has a shell	is small	used for protection
silicon	found in sand	found in rocks	used in computers
silicone	a liquid	is clear	used in cooking
silk	is shiny	is smooth	a fabric
silver	is shiny	a metal	is white
skin	covers the body	has hair	is thin
slate	used for writing on	a rock	is black

smoke	comes from fire	a gas	is white
snow	is white	is cold	falls from the sky
soap	is white	used for cleaning	used for washing
soil	used for growing plants	is brown	used for growing crops
spandex	is stretchy	used for making clothes	is elastic
sponge	used for cleaning	has holes	is soft
steel	is strong	is hard	is shiny
straw	used for making baskets	used for making hats	used for making brooms
suede	is soft	is brown	made of leather
sugar	is sweet	is white	has calories
sulfur	is yellow	found in rocks	found in volcanoes
talc	is soft	is white	a mineral
talcum powder	is white	used for babies	used on babies
tar	is sticky	is black	used to make roads
tarpaper	made of wood	has a rough surface	is thin
teakwood	used for furniture	is hard	used for flooring
teflon	is slippery	used for cooking	used for frying
terracotta	used for making pots	is brown	made of clay
tin	is shiny	a metal	made of metal
tinfoil	is shiny	is thin	is recycled
tinsel	is shiny	made of metal	used for decorating christmas trees
titanium	is strong	a metal	is light

toilet paper	is white	is soft	used for cleaning
tooth	is white	is hard	has a cavity
toothpaste	is white	used for cleaning teeth	has fluoride
topaz	is yellow	a gemstone	a mineral
tungsten	a metal	used in light bulbs	is hard
tweed	a fabric	is brown	made of wool
uranium	is radioactive	a metal	found in the ground
vapor	is invisible	a gas	made of water
vaseline	is white	used as a lubricant	used for lips
velcro	is sticky	used for shoes	used for clothing
velvet	is soft	a fabric	is shiny
vinyl	used for records	a type of plastic	used for making records
water	is clear	is wet	a liquid
wax	is sticky	is white	used for candles
wax paper	is thin	is white	used for wrapping food
wool	is soft	is warm	comes from sheep
zinc	a metal	is shiny	is white
zirconium	a metal	is hard	is shiny

Table S5. Descriptive labels of all 36 model dimensions obtained from GPT-3 feature norms.

dimension (label)	Top 3 features (weights on dimensions)		
1 (mineral)	is hard (2.29)	a metal (1.98)	a mineral (1.60)
2 (wood)	used for making furniture (3.74)	a wood (2.07)	is hard (1.81)
3 (metallic)	a metal (5.00)	is shiny (4.30)	is hard (1.81)
4 (fabric)	used for making clothes (4.33)	is soft (3.46)	a fabric (2.79)
5 (white)	is white (6.39)	is soft (1.38)	is thin (1.16)
6 (grainy)	is black (1.05)	is grey (0.79)	used for making roads (0.79)
7 (crystalline)	a gemstone (3.94)	is shiny (3.56)	used for making jewelry (3.02)
8 (fibrous)	is green (1.73)	has leaves (1.38)	a plant (1.31)
9 (cloudy)	a gas (1.60)	is hot (1.38)	is cold (1.23)
10 (small)	is hard (1.62)	is shiny (1.54)	used for making jewelry (1.53)
11 (beige, tan color)	is yellow (1.39)	is brown (1.06)	used for making furniture (0.88)
12 (viscous)	is sticky (2.76)	a liquid (2.20)	is clear (1.00)
13 (black)	is black (5.86)	is sticky (0.93)	used for writing (0.91)
14 (brick)	is hard (1.53)	made of clay (1.12)	is rectangular (1.00)
15 (swirly)	used for making clothes (2.31)	is soft (2.05)	a fabric (1.67)
16 (maroon)	used for making furniture (2.74)	a wood (1.83)	is brown (1.80)
17 (lines, long, vertical, tubular)	used for making furniture (2.01)	a metal (1.97)	is hard (1.61)
18 (mesh)	is strong (2.44)	is metallic (1.56)	is heavy (1.12)
19 (yellow)	is yellow (3.83)	is poisonous (1.02)	a plant (0.96)

20 (multi-colored)	is colorful (2.38)	is white (1.55)	is soft (1.29)
21 (thin)	is thin (4.26)	is white (2.11)	used for writing (2.00)
22 (bulbous)	is white (2.50)	is soft (2.06)	a gas (1.33)
23 (green)	is green (7.50)	a plant (3.52)	has leaves (2.81)
24 (blue)	is blue (1.92)	is transparent (1.55)	a gemstone (1.19)
25 (shell, bone)	is white (4.64)	is hard (3.72)	made of calcium (1.30)
26 (gemstone)	used for making jewelry (3.86)	is shiny (3.73)	a gemstone (2.60)
27 (sheet)	is thin (4.13)	made of paper (2.35)	used for wrapping food (2.09)
28 (hair)	is soft (3.72)	used for making clothes (2.34)	is warm (1.46)
29 (spongy)	used for cleaning (1.91)	is white (1.54)	is thin (1.34)
30 (golden, shiny)	is shiny (5.87)	a metal (5.39)	is metallic (1.84)
31 (red)	is red (2.96)	a gemstone (1.69)	is hot (1.51)
32 (round)	made of clay (3.11)	is white (1.73)	is fragile (1.37)
33 (cream color)	is white (3.15)	made of wood (1.01)	used for writing (0.69)
34 (bumpy)	is soft (1.20)	has bubbles (1.18)	is sweet (0.98)
35 (hot)	is hot (7.41)	is red (4.76)	is dangerous (2.71)
36 (turquoise)	is soft (2.00)	is clear (1.46)	is smooth (1.43)

SI References

1. M. N. Hebart, A. H. Dickter, A. Kidder, W. Y. Kwok, A. Corriveau, C. van Wicklin, C. I. Baker, THINGS: A database of 1,854 object concepts and more than 26,000 naturalistic object images. *PLoS One* 14:e0223792 (2019).
2. C. Y. Zheng, F. Pereira, C. I. Baker, M. N. Hebart, Revealing interpretable object representations from human behavior. *Int. Conf. Learn. Represent.* (2019).
3. M. N. Hebart, C. Y. Zheng, F. Pereira, C. I. Baker, Revealing the multidimensional mental representations of natural objects underlying human similarity judgements. *Nat. Hum. Behav.* 4, 1173–1185 (2020).
4. D. P. Kingma, J. Ba, Adam: A Method for Stochastic Optimization. *arXiv* 1412.6980 (2014).
5. Princeton University, About WordNet. *WordNet* https://wordnet.princeton.edu/ (2010).
6. T. B. Brown, et al., Language Models are Few-Shot Learners. *Adv. Neural. Inf. Process. Syst.* 33 (2020).
7. H. Hansen H., M. N. Hebart, Semantic features of object concepts generated with GPT-3. *arXiv* 2202.03753 (2022).
8. G. Salton, C. Buckley, Term-weighting approaches in automatic text retrieval. *Inf. Process. Manag.* 24, 513–523 (1988).
9. M. N. Hebart, et al., THINGS-data, a multimodal collection of large-scale datasets for investigating object representations in human brain and behavior. *Elife* 12, e82580 (2023).
10. P. Kaniuth, F. P. Mahner, J. Perkuhn, M. N. Hebart, A high-throughput approach for the efficient prediction of perceived similarity of natural objects. *bioRxiv* (2024).
11. M. T. Pilehvar, N. Collier,. *arXiv* 1608.01961 (2016).
12. T. Mikolov, I. Sutskever, K. Chen, G. Corrado, J. Deank, Distributed Representations of Words and Phrases and their Compositionality. *arXiv* 1310.4546 (2013).
13. Z. Wu, M. Palmer, Verb Semantics and Lexical Selection. *Proc. Conf. Assoc. Comput. Linguist.* (1994).
